# Characterization and prognostic of CD8 + TIM3 + CD101 + T cells in glioblastoma multiforme

**DOI:** 10.1186/s13578-025-01390-1

**Published:** 2025-05-15

**Authors:** Hong-Liang Wang, Sai Li, Chun-Chun Ma, Xiang-Hu Zheng, Hao-Yuan Wu, Chen-Xi Chang, Zhi-Hao Yang, Jia-Wei Wang, Fa-Ming Pan, Bing Zhao

**Affiliations:** 1 Department of Neurosurgery, The Second Affiliated Hospital of Anhui Medical University, No 678 Furong Road, Hefei Economic and Technological Development Zone, Hefei, 230000 People’s Republic of China; 2 Department of Neurosurgery, National Cancer Center/National Clinical Research Center for Cancer/Cancer Hospital, Chinese Academy of Medical Sciences and Peking Union Medical College, No 17 Panjiayuan Nanli, Chaoyang District, Beijing, 100021 People’s Republic of China; 3 Department of Epidemiology and Biostatistics, School of Public Health, Anhui Medical University, 81 Meishan Road, Hefei, 230032 Anhui China; 4 The Key Laboratory of Major Autoimmune Diseases, Anhui Medical University, 81 Meishan Road, Hefei, 230032 Anhui China

**Keywords:** Glioblastoma multiforme, CD8 + TIM3 + CD101 + T cells, Prognostic model, Single- cell RNA sequencing, Immune microenvironment

## Abstract

**Background:**

Glioblastoma multiforme (GBM) is a pervasive and aggressive malignant brain tumor. In the tumor immune microenvironment, CD8 + TIM3 + CD101 + T cells (CCT cells) play a pivotal role in tumor progression and immune evasion. This study aimed to characterize differentially expressed genes (DEGs) in CCT cells, establish a prognostic model for GBM, and explore clinical implications.

**Methods:**

Analysis of data from TCGA, CGGA, and GEO databases included whole-genome expression profiles, clinical data, single nucleotide mutations, and single-cell RNA sequencing. DEGs were identified, and cell trajectories were constructed using Seurat, Monocle 2, and CellChat packages. Functional enrichment analysis was conducted with clusterProfiler, and a prognostic model was developed. Immune infiltration and drug sensitivity analyses were performed to evaluate therapeutic implications.

**Results:**

Eight distinct cell types were distinguished, encompassing T cells, macrophages, neurons, mural cells, endothelial cells, oligodendrocytes, fibroblasts, and B cells. Comparative analysis revealed differences in these cell types between GBM samples with new adjuvant therapy and initial diagnosis controls. Pseudotime analysis indicated CD8 + TIM3 + CD101-T cells as precursors to CCT cells, unveiling unique gene expression patterns during this transition. The prognostic model, incorporating 22 gene features via LASSO regression, demonstrated strong predictive ability through Receiver Operating Characteristic (ROC) curves. Analysis of 28 immune cell types revealed differences between high-risk and low-risk groups, providing insights into GBM’s immune evasion mechanisms. Drug sensitivity analysis proposed potential therapeutic strategies for high-risk patients.

**Conclusion:**

This study offers an in-depth understanding of CCT cells in GBM, introducing a novel prognostic model and suggesting promising therapeutic approaches.

**Supplementary Information:**

The online version contains supplementary material available at 10.1186/s13578-025-01390-1.

## Introduction

Glioblastoma multiforme (GBM) is the most aggressive, malignant, and fatal of the central nervous system tumors [1, 2]. Due to the tumor heterogeneity, suppressive tumor microenvironment, low immunogenicity, infiltration of tumor stem cells, and limitations imposed by the blood-brain barrier, standard treatment approaches including surgery, radiation, chemotherapy, as well as newer treatment options such as immunotherapy, electric field therapy, have limited efficacy against GBM [3]. Despite the implementation of aggressive treatment protocols, the median survival duration for the majority of patients extends merely to 12–15 months. Further, less than 5% of patients survive five years [4, 5]. In recent years, there has been increasing interest in the use of PD-1 blockade therapy for GBM. PD-1 is an immune checkpoint protein located on the surface of T cells, while its ligand PD-L1 is primarily expressed on tumor cell surfaces. The binding of PD-L1 to PD-1 inhibits T cell activity, allowing tumor cells to evade immune system surveillance and attack [6]. Currently, PD-1 blockade therapy shows significant potential in cancer treatment, markedly improving the prognosis of many patients. However, a portion of patients does not benefit from this treatment, and the underlying mechanisms for this phenomenon remain unclear. Consequently, further research and more targeted therapeutic strategies are needed to enhance the prognosis of GBM patients.

Recently, there has been a growing focus on the influence of the glioma immune microenvironment, encompassing glioma cells, stromal cells, and immune cells, on the initiation, advancement, and immune escape of GBM [7, 8]. Varied compositions of immune cells, including T cells and their distinct subpopulations, serve as key determinants influencing the immunosuppressive milieu [9–11]. Recent study has identified a specific T cell subset among various immune cell populations—CD8 + TIM3 + CD101 + T cells (referred to as CCT cells)—that is closely associated with the outcomes of PD-1 blockade therapy. Originally identified in a mouse model of chronic viral infection and classified as exhausted T cells after chronic viral infection, CCT cells represent a subset of cells with CD8 + CD101 + TIM3 + phenotype [12, 13]. Among patients with non-small cell lung cancer, an elevated proportion of this subgroup is significantly linked to adverse reactions to immunotherapy, potentially due to reduced migratory and functional capabilities [14]. Following in vitro stimulation, the cytokine levels in CCT cells from responders were significantly higher than those from non-responders, suggesting that these factors may be associated with differences in treatment outcomes. GBM presents a unique immunosuppressive microenvironment, where tumor-associated macrophages (TAMs) play a crucial role. These TAMs interact with T cells, undermining their antitumor activity and potentially promoting tumor growth, thus limiting the effectiveness of PD-1 blockade therapy [15]. Therefore, investigating the dynamic changes and underlying mechanisms of CCT cells in the context of PD-1 blockade therapy in GBM is of significant research value and could provide opportunities for more patients to benefit.

In this investigation, we employed sophisticated bioinformatics techniques such as single-cell RNA sequencing data analysis, pseudotime analysis, cell communication analysis, gene enrichment analysis, and the construction of prognostic models. Our study sheds light on the prognostic relevance of CCT cell-related DEGs in GBM and their impact on the immune microenvironment. These discoveries may aid in the formulation of enhanced therapeutic approaches and the advancement of prognostic outcomes for GBM patients.

## Materials and methods

### Data download and analysis for routine transcriptomes

All data utilized in this study are publicly available, primarily sourced from The Cancer Genome Atlas (TCGA) database (https://portal.gdc.cancer.gov/) and the Chinese Glioma Genome Atlas (CGGA) database (https://www.cgga.org.cn/). Utilizing the R package “TCGAbiolinks” (version 2.25.0) [16], we obtained genome-wide expression profiles for GBM in TPM format, clinical data, and single nucleotide mutation data (SNV) predicted by the VarScan2 Variant Aggregation and Masking tool from the TCGA database. The TCGA-GBM cohort (*n* = 175) comprised 170 tumor samples and 5 normal control samples. Of these, 159 glioma tumor samples possessed associated survival data and were incorporated into the present study. Additionally, expression profiles and clinical data from the CGGA-mRNAseq-693 and CGGA-mRNAseq-325 cohorts, as obtained from the CGGA database, were incorporated into this study. The CGGA-mRNAseq-693 dataset comprises 693 samples, while the CGGA-mRNAseq-325 dataset includes 325 samples. From these, we selected 139 glioblastoma multiforme (GBM) samples with a World Health Organization (WHO) Grade 4 classification from the CGGA-mRNAseq-325 dataset, and 249 GBM samples with a WHO Grade 4 classification from the CGGA-mRNAseq-693 dataset. In total, 374 glioma tumor samples had complete survival information.

### Download and analyze single-cell RNA sequencing data

In this investigation, the single-cell RNA sequencing dataset GSE235676, which encompassing glioblastoma neoadjuvant therapy, newly diagnosed glioblastoma samples, and controls was obtained from the Gene Expression Omnibus (GEO) database (https://www.ncbi.nlm.nih.gov/geo/). Specifically, GSE235676 consisted of 5 glioblastoma samples treated with TMZ + RT, 7 newly diagnosed glioblastoma samples, and 12 neoadjuvant glioblastoma samples (anti-PD-1 therapy). For analysis, 7 newly diagnosed glioblastoma samples and 12 neoadjuvant glioblastoma samples were selected. The Seurat package in R was used to analyze the single-cell raw data from GSE235676 [17]. The following criteria were used to filter low-quality cells and genes: [1] Genes with undetected expression were excluded; [2] Cells exhibiting expression of more than 50 genes were retained; [3] Cells with UMI counts below 30,000 were retained; [4] Cells retaining less than 20% of their mitochondrial genes were included; [5] Cells with more than 0.75 genes detected per UMI were retained; [6] Cells retaining less than 1% of their red blood cell genes were included. Following data standardization, identified highly variable genes in single cells by balancing average expression with dispersion. Following principal component analysis (PCA), the significant principal components (PCs) were used as input for graph-based clustering. By using the harmony method, the batch effect of different samples was eliminated. For the clustering analysis, we employed the FindClusters function, which generated 32 clusters from 20 PCs at a resolution parameter of 0.6. We then performed unified manifold approximation and projection (UMAP) using the RunUMAP function. UMAP-1 and UMAP-2 were utilized to demonstrate cellular clustering. We then used the FindAllMarkers function to identify the differential genes in each cell population. Following this, cell clusters were identified using cell type-specific biomarkers, and the proportions of different types of cells were calculated.

### Cell trajectories were delineated through pseudotime ordering analysis

Monocle 2 (version 2.22.0) was used to perform the pseudotime analysis [18]. By estimating the size factor of trajectory inference, the raw count data of CCT precursor cells and CCT cells was normalized for pseudotime analysis. A pseudo-temporal trajectory [19] was constructed using genes that are highly discrete and expressed (discrete estimate ≥ 1, average expression ≥ 0.1). A default set of parameters was applied to DDRTree algorithm 1. To delve deeper into these branching events, Branch Expression Analysis Modeling implemented in Monocle 2 was utilized, aiding in the identification of all significantly branch-dependent gene expressions [18]. Monocle 2 was employed to visualize the branch-dependent expression pattern as a heatmap.

### Cell-to-cell communication analysis and ligand-receptor expression

According to the “CellChat” (version 1.1.3) R software package, CellChat objects were generated based on UMI counts matrices for each group (neoadjuvant therapy and newly diagnosed group) (http://www.github.com/sqjin/CellChat)(20) [20]. In this study, cell-to-cell communication was analyzed utilizing the default parameters of the “CellChatDB.human” ligand-receptor interaction database. In order to compare the interaction counts and strengths between groups, CellChat objects from each group were merged using the “mergeCellChat” function. NetVisual_diffInteraction was used to visualize differences in interaction quantity or intensity between different types of cells within groups. Lastly, we visualized intergroup signaling gene expression by using “netVisual_bubble” and “netVisual_aggregate”.

### Analysis of GO and KEGG pathway enrichment

The Gene Ontology (GO) and Kyoto Encyclopedia of Genes and Genomes (KEGG) enrichment analysis of differentially expressed genes in GBM was conducted using ClusterProfiler [21].

### Prognostic model development and validation

To study whether differentially expressed genes in CCT cells in glioblastoma correlate with overall survival (OS), we used univariate Cox regression analysis. A 7:3 ratio was used to divide all tumor samples with clinical information into training (*n* = 364) and validation (*n* = 170). In order to narrow down the number of candidate genes and construct a prognostic model, a LASSO Cox regression model (R package “glmnet” (version 4.1.8)) was used [22]. Based on the minimum criterion, the penalty parameter (λ) was determined. The following formula was used to calculate the risk score:

$${\rm{riskScore}} = \sum\limits_{{\rm{i}} = 1}^{\rm{n}} {{\rm{Coef}}\left( {{\rm{gen}}{{\rm{e}}_{\rm{i}}}} \right)*{\rm{Expression}}\left( {{\rm{gen}}{{\rm{e}}_{\rm{i}}}} \right)} $$

Training set samples were divided into low-risk and high-risk groups using the surv_cutpoint function. Kaplan-Meier survival curves were plotted to evaluate prognostic value, and the log-rank test was applied to assess statistical significance. Based on Receiver Operating Characteristic curves (ROC), prognostic models were validated. The Area Under the Curve value, which generally ranges from 0.5 to 1, signifies that a value approaching 1 denotes superior model performance. In the validation set, the same values were used as in the training set to divide the samples into low-risk and high-risk groups. We also considered Youden’s index, a statistical indicator used to assess the validity of diagnostic tests. It is particularly useful for determining the optimal cut-off point to distinguish between two groups. Youden’s index is calculated as: J = Sensitivity + Specificity − 1, where Sensitivity is the true positive rate (TPR) and Specificity is the true negative case rate (TNR), i.e., 1 minus the false positive rate (FPR). Youden’s index can range from − 1 to 1, with closer to 1 indicating better performance of the model.

### Nomogram construction and validation

From the TCGA and CGGA cohorts, clinical information (gender and age) was extracted. Clinical data were combined with the risk scores from the prognostic model for univariate and multivariate Cox regression analyses. In addition, the “RMS” (version 6.3.0) package in R was used to construct a nomogram integrating prognostic and clinical features. The nomogram was evaluated through time-dependent ROC curve analysis.

### Gene set enrichment analysis (GSEA)

In this research, differential expression analysis between the high-risk and low-risk groups was performed using the R package “limma” (version 3.50.3) [23]. GSEA, performed through the “clusterProfiler” (version 4.2.2) package in R.

### Gene set variation analysis (GSVA)

GSVA was used to investigate biological functional variances between high-risk and low-risk groups using the R package “GSVA” (version 1.42.0).

### Immune infiltrations analysis

Single-sample gene set enrichment analysis (ssGSEA) calculates enrichment scores separately for each sample and gene set in GSEA. Using data from the TISIDB (Tumor and Immune System Interactions Database) source, comprising 28 immune cell types, relative enrichment scores were calculated for each immune cell based on the gene expression profiles of individual tumor samples.

### Somatic mutations analysis

Based on mutation data, a landscape of genomic mutations was depicted. The R package “maftools” (version 2.10.5) was used to visualize somatic alterations within distinct clusters, including single nucleotide polymorphisms, insertions and deletions, tumor mutation burdens (TMBs), and mutation frequencies [24]. Mutation frequency analysis shows that the top 20 frequently mutated genes are key tumor drivers [25].

### Drug sensitivity analysis

In this study, “oncoPredict” (version 1.2) [26] was utilized to analyze and predict potential therapeutic drug sensitivities for patients in different risk groups with GBM using IC50 data and corresponding gene expression data obtained from the Genomics of Drug Sensitivity in Cancer (GDSC) database (https://www.cancerrxgene.org/)(27)[27].

### Immune checkpoints

In this study, immune checkpoint genes (ICGs) were compared between high-risk and low-risk groups.

### Tumor immune dysfunction and exclusion (TIDE)

TIDE (http://tide.dfci.harvard.edu/) analysis was conducted to evaluate the immune responses of patients in this study.

### Statistical analysis

Data processing and statistical analyses were conducted using R (version 4.1.2). To compare survival rates between two groups, Kaplan-Meier curves along with log-rank tests were employed. All survival curves were generated with the “survminer” (version 0.4.9) package in R. Univariate and multivariate Cox regression analyses were carried out to assess prognostic variables, while Lasso regression was applied to identify significant outcome-related factors. Data visualization was performed using the “ggplot2” (version 3.5.0) package, and overall survival (OS) and risk scores were calculated using the “survival” package. Heatmaps were created with the “Pheatmap” (version 1.0.12) package. The significance of quantitative differences in normally distributed variables was analyzed using two-tailed t-tests or one-way ANOVA, whereas Wilcoxon or Kruskal-Wallis tests were used for non-normally distributed data. All statistical analyses were executed in R software, and a P-value of less than 0.05 was considered statistically significant.

## Results

### Clustering, dimension reduction and cell annotation of single-cell RNA sequencing data

**Fig. 1:**
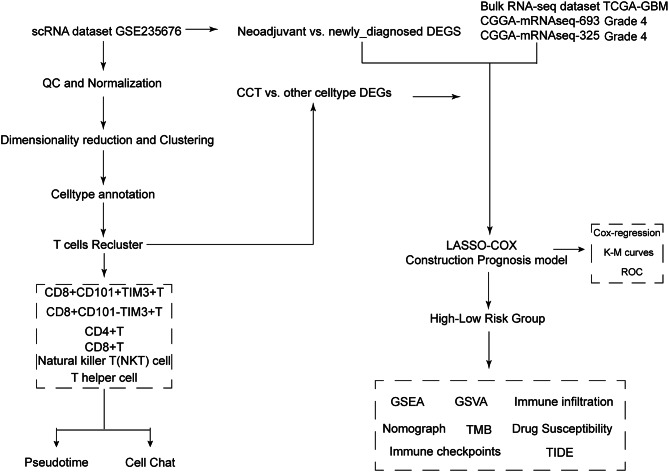
Flow chart of this study

**Fig. 2:**
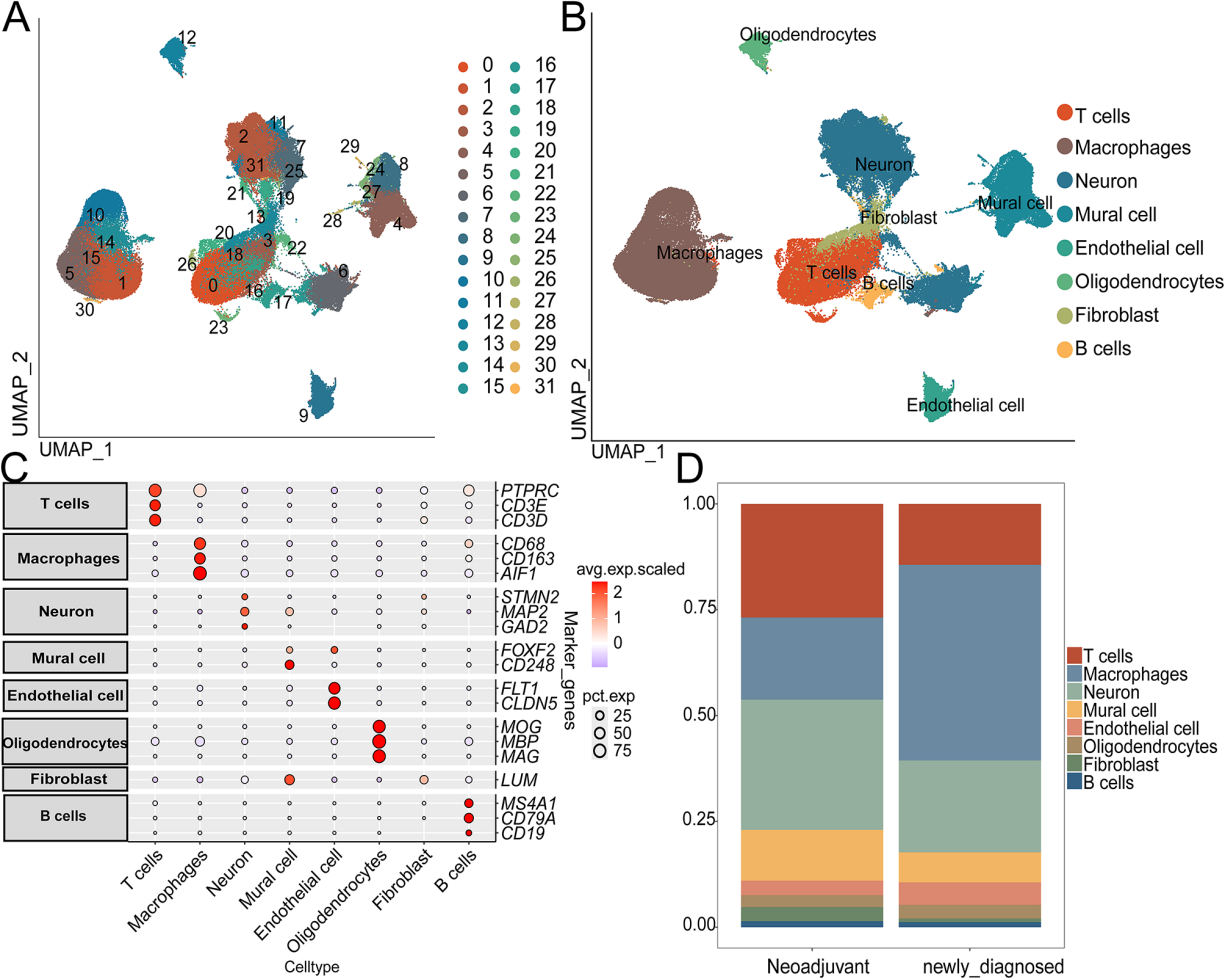
Cell subpopulations were identified using single-cell sequencing. (**A**) The UMAP plot displays the distribution of subclusters of glioblastoma cells. (**B**) The UMAP plot illustrates the annotation results of subclusters of glioblastoma cells. (**C**) The expression of marker genes in each cell type. (**D**) The stacked bar plot shows the distribution of all cell types in each sample

Figure 1 shows the workflow of this study. A total of 113,446 cells were obtained from the single-cell transcriptome data GSE235679. Figure 2A illustrates the clustering of all cells into 32 distinct groups, with cell types identified using cell-specific biomarkers based on gene expression characteristics (Table S1). Figure 2B displays 8 identified cell types (T cells, Macrophages, Neuron, Mural cell, Endothelial cell, Oligodendrocytes, Fibroblast, and B cells). Figure 2C presents dot plots of characteristic genes for each cell type, and Fig. 2D, along with Table S2, displays cumulative histograms of cell type proportions across groups. We observed a significant increase in the proportion of T cells within the tumors of patients following PD-1 blockade therapy, while the number of macrophages markedly decreased. This reflects an improvement in the immune microenvironment and a reduction in immune suppression induced by tumor-associated macrophages (TAMs), suggesting enhanced immune surveillance and attack capability against the tumor. This change may be associated with a favorable response of patients to PD-1 blockade therapy.

### Annotating CCT cells

**Fig. 3:**
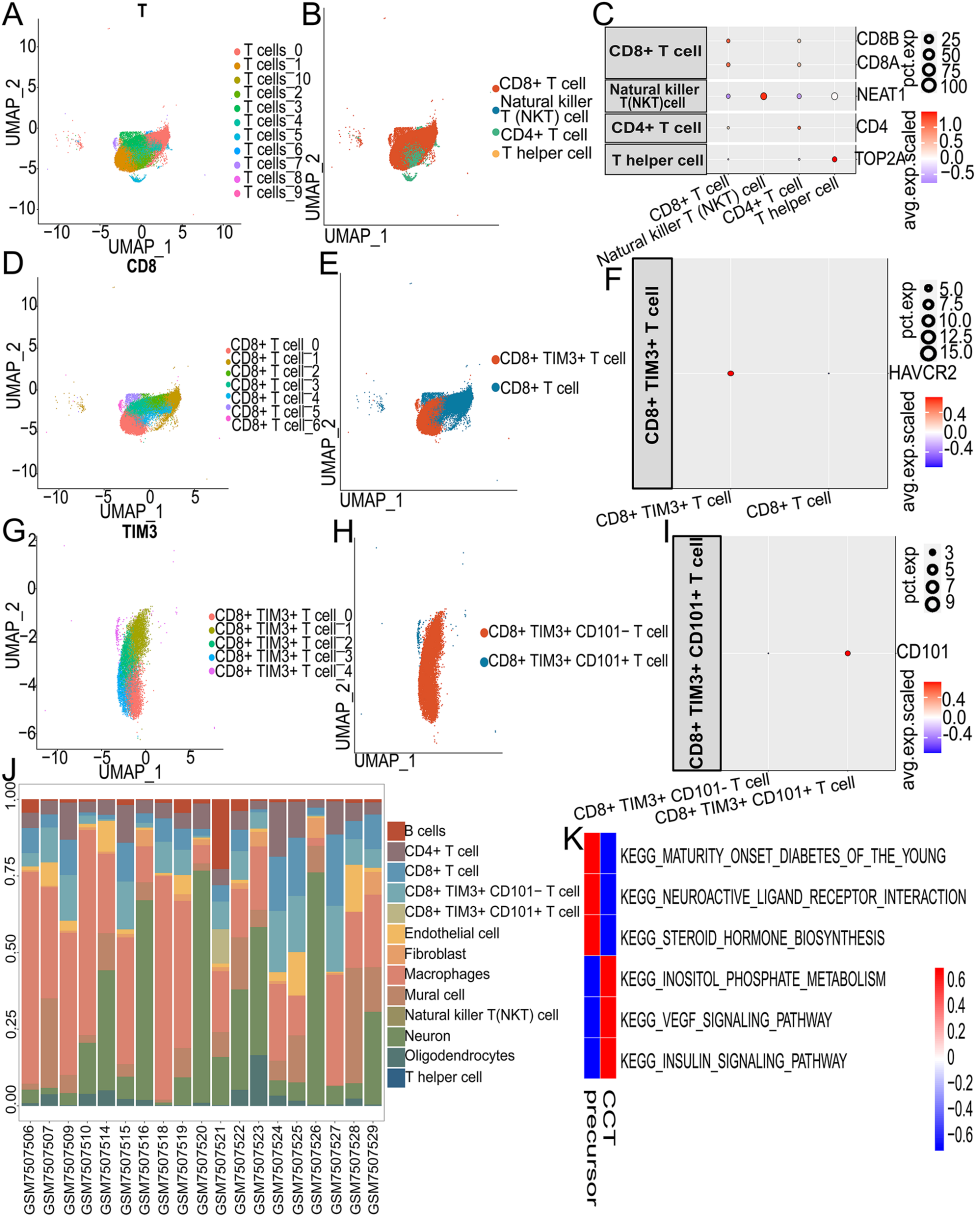
Annotation of T cell subpopulations. (**A**) The UMAP plot displays the distribution of T cell subtypes. (**B**) The UMAP plot shows the annotation results of T cell subtypes. (**C**) Expression of marker genes in each cell type. (**D**) The UMAP plot illustrates the distribution of CD8 + T cell subtypes. (**E**) The UMAP plot depicts the annotation results of CD8 + T cell subtypes. (**F**) Expression of marker genes in each cell type. (**G**) The UMAP plot showcases the distribution of CD8 + TIM3 + T cell subtypes. (**H**) The UMAP plot visualizes the annotation results of CD8 + TIM3 + T cell subtypes. (**I**) Expression of marker genes in each cell type. (**J**) Stacked bar graph showing the proportions of different cell subtypes in each sample. (**K**) Heatmap displaying the top 3 pathways most significantly enriched in CCT cell subtypes

We extracted T cells and annotated them with improved resolution (Fig. 3A). In Fig. 3B, four types of T cells were identified: CD4 + T cells, CD8 + T cells, Natural killer T cells, and T helper cells. The dot plot (Fig. 3C) shows the specific genes of each cell type. Based on expression of HAVCR2, we identified CD8 + T cells as CD8 + TIM3 + T cells and CD8 + T cells (Fig. 3D-F). Finally, based on the expression of the CD101 gene, CD8 + TIM3 + T cells were further categorized into CD8 + TIM3 + CD101- T cells (CCT precursor cells) and CD8 + TIM3 + CD101 + T cells (CCT cells) (Fig. 3G-I). Stacking bar graphs illustrate the proportion of different kinds of cells in each sample (Fig. 3J, Table S3). Additionally, pathways from the MsigDB database were used to perform GSVA on CCT and CCT precursor cells (Table S4). A heatmap illustrating pathway activities was generated, highlighting the top three pathways with the most significant differences among CCT cell subgroups (Fig. 3K).

### Pseudotime analysis

**Fig. 4:**
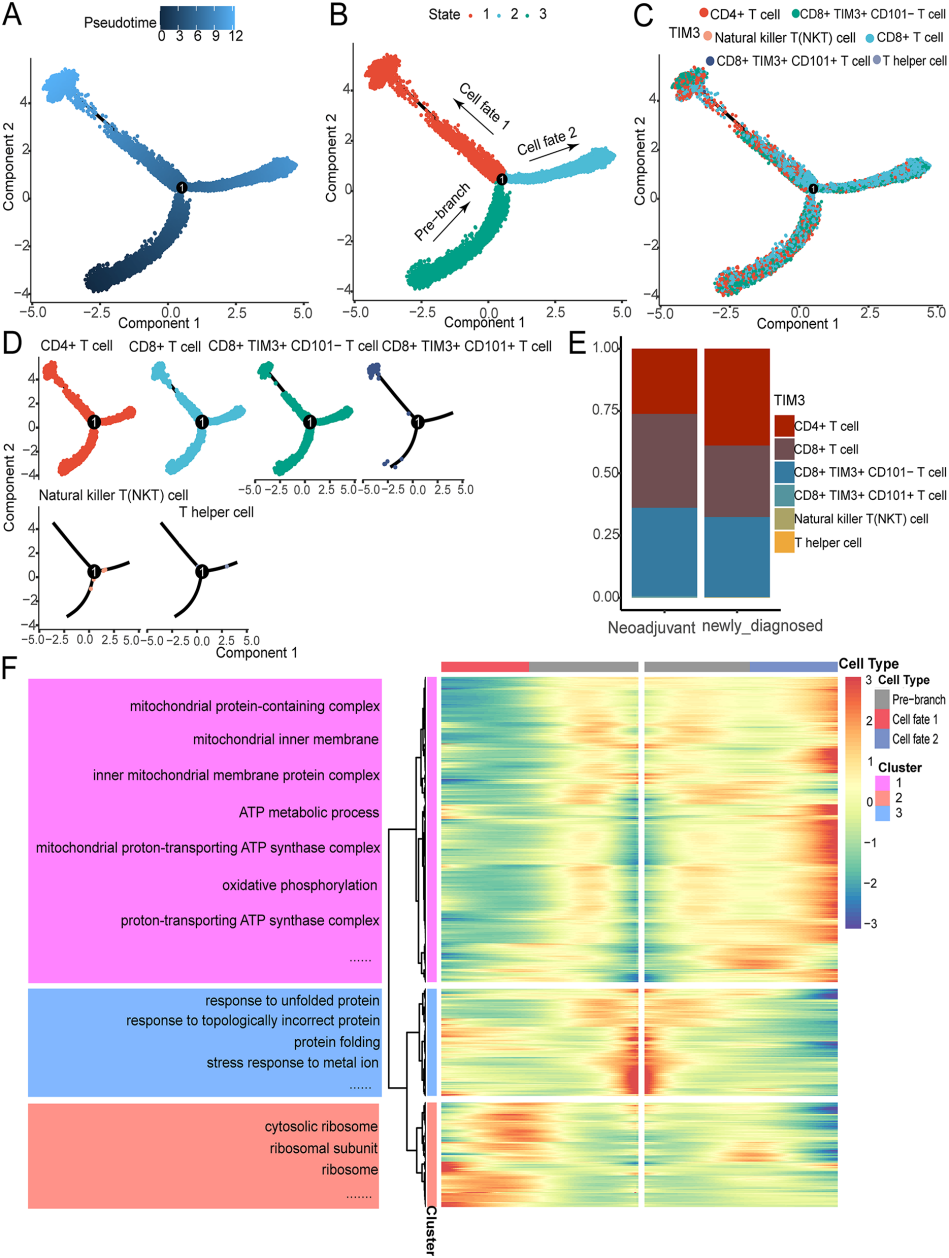
Pseudotime analysis. (**A**) The pseudotime coloring transitions from deep blue to light blue. (**B**) The pseudotime trajectory is segmented into three distinct states by Monocle2. (**C**) Pseudotime trajectory illustrating the distribution of glioblastoma cells based on cell types. (**D**) Pseudotime trajectory showing the distribution of glioblastoma cells based on cell type grouping. (**E**) Stacked bar graph displaying the distribution of cell types across different groups. (**F**) Heatmap depicting the differentially expressed genes (DEGs) in various branches (cell fates). Enriched GO pathways for different gene clusters in the heatmap are shown on the left

To identify fundamental gene expression programs dictating glioblastoma progression, we constructed a pseudo-temporal cell trajectory based on T cell subtypes. The transcriptional states along the trajectory indicate distinct processes. As shown in Fig. 4A-D, CD8 + TIM3 + CD101 + T cells are primarily located at the end of the state, while CD8 + TIM3 + CD101- T cells, which serve as precursors to CD8 + TIM3 + CD101 + T cells, can be found across various states. Notably, state 2 predominantly contains CD8 + TIM3 + CD101- T cells associated with favorable outcomes in immunotherapy, indicating that this differentiation pathway is a key factor in the effectiveness of immunotherapy. A bar graph illustrates the proportions of T cell subtypes across different groups (Fig. 4E). Our study investigated the genes influencing glioblastoma cell branching patterns to elucidate the molecular basis of transformation. The results (Fig. 4F, Table S5) show that four pathway-related genes, associated with “response to unfolded proteins” and “response to topologically incorrect proteins”, were highly expressed in the pre-branch stage. Genes linked to the “mitochondrial protein-containing complex” and two other pathways showed increased expression in cell branch 1. Genes associated with four pathways, including “antigen processing and presentation” and “positive regulation of cytokine production”, were significantly expressed in cell branch 2.

### Cell-to-cell communication analysis

**Fig. 5:**
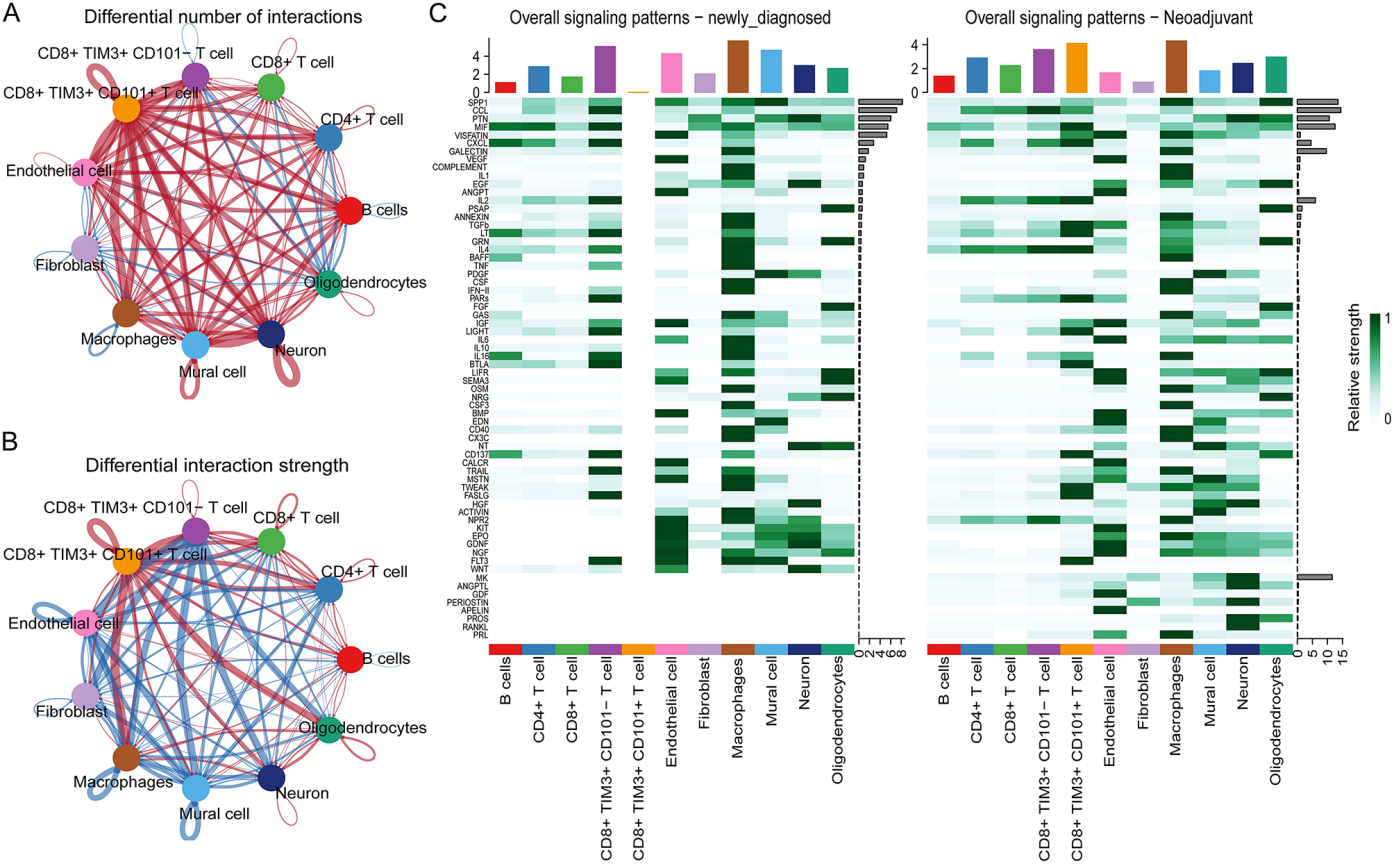
Displays the quantity and intensity of cell communication between the newly diagnosed and neoadjuvant therapy groups. (**A**) The network graph illustrates the variation in the number of interactions between cell types in the newly diagnosed and neoadjuvant therapy groups. The red line indicates the newly diagnosed group, while the blue line represents the neoadjuvant therapy group. The thickness of the lines represents changes in the number of interactions between cell types, with thicker lines indicating a greater number of interactions between the two cell types. (**B**) The network graph depicts the change in interaction intensity between cell types in the newly diagnosed and neoadjuvant therapy groups. The red line indicates the newly diagnosed group, while the blue line represents the neoadjuvant therapy group. The thickness of the lines reflects the strength of the interaction, with thicker lines indicating a stronger interaction between the two cell types. (**C**)The heatmap portrays the overall signaling patterns for the newly diagnosed and neoadjuvant therapy groups

**Fig. 6:**
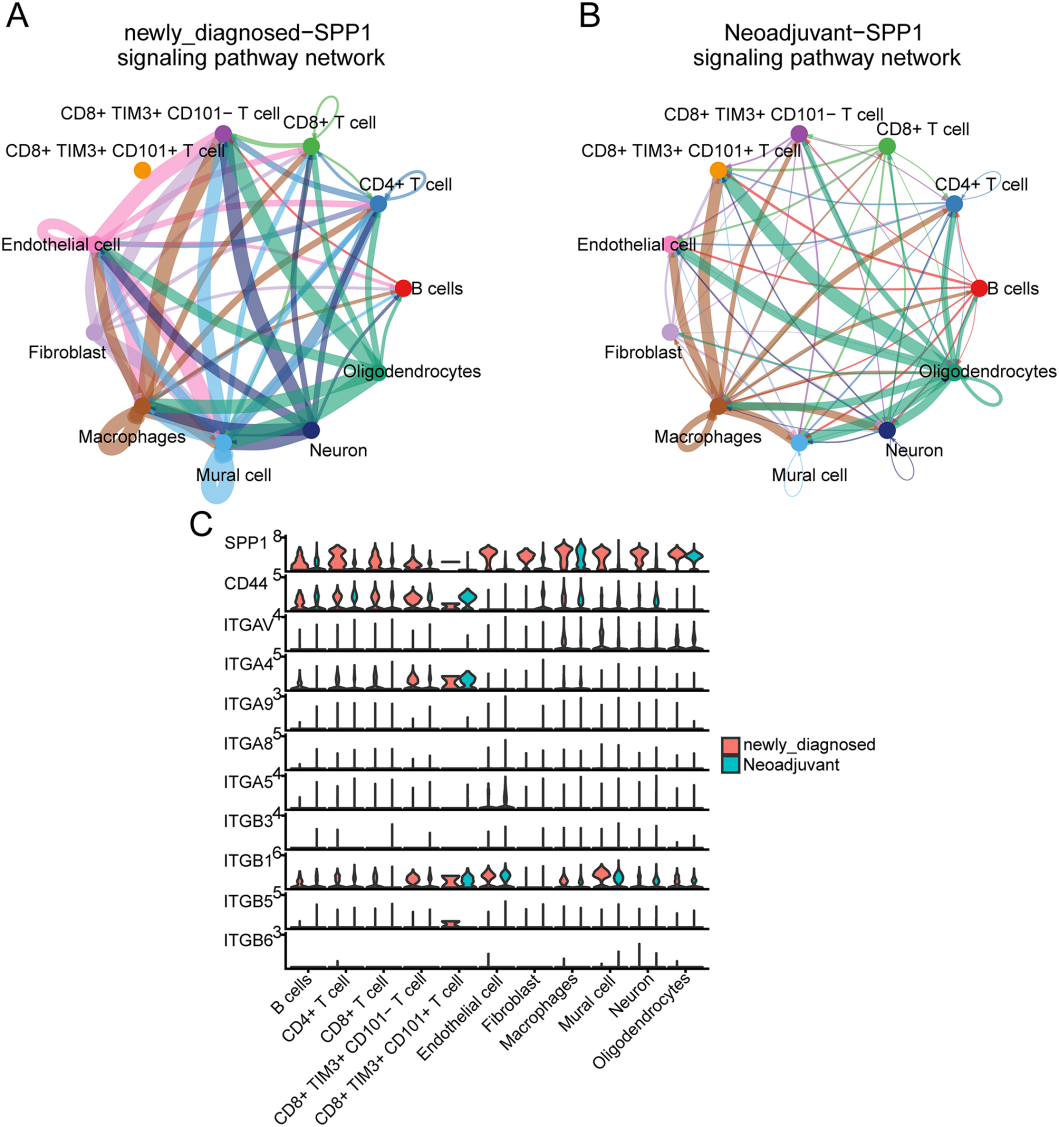
The network diagram illustrates the intercellular communication signals emitted by each cell type in the newly diagnosed (**A**) and neoadjuvant therapy (**B**) groups, with different line colors corresponding to different cell types. The thickness of the lines represents the strength of the signals, with thicker lines indicating a stronger interaction between the two cell types. (**C**) The violin plot displays the expression levels of all ligands and receptors in the SPP1 signaling pathway across different cellular clusters

Intercellular communication is a crucial component of single-cell research. These interactions are shown in Fig. 5A and B. Given the significant role of CD8 + TIM3 + CD101- T cells in PD-1 blockade therapy, it is essential to explore their cellular interactions in greater depth. These interactions may represent the primary factor driving the differentiation of CD8 + TIM3 + CD101- T cells towards a terminal stage and are believed to be critical in influencing the outcomes of immune therapies. As shown in Fig. 5C, the signaling pathways involving CD8 + TIM3 + CD101- T cells primarily focus on SPP1, CCL, PTN, MIF, and VISFATIN. However, these signals do not differentiate whether CD8 + TIM3 + CD101- T cells are the initiators or recipients of the signaling, which is important for understanding the function of this cell subset. This distinction is crucial because signals received by CD8 + TIM3 + CD101- T cells may be the key mediators of their terminal differentiation. Therefore, we performed a cell communication analysis between the PD-1 treated and untreated groups to identify communication signals in which CD8 + TIM3 + CD101- T cells act as recipients, and where significant changes in signaling were observed across groups. Notably, we found that SPP1 and CCL exhibited the most significant inter-group differences in signal strength, with a particularly strong alteration in SPP1 signaling, which attracted further attention from us (Supplementary Fig. 1 ). We visualized the communication strength of the SPP1 pathway between newly diagnosed patients and those undergoing neoadjuvant therapy and found that CD8 + TIM3 + CD101- T cells received SPP1 signals from multiple cell types, with a marked decline in signal strength after PD-1 blockade therapy (Figs. 6A-B). These results provide initial insights into the potential interactions between these cell types, aiding in further research on the comprehensive actions of CCT cells in glioblastoma. Figure 6C illustrates high expression of the receptor in the SPP1 signaling pathway in CCT cells under neoadjuvant therapy. Targeting SPP1 may offer significant therapeutic benefits for patients, which could represent a pivotal factor influencing the efficacy of PD-1 blockade therapy.

### Enrichment analysis of CCT-related genes with differential expression in glioblastoma

**Fig. 7:**
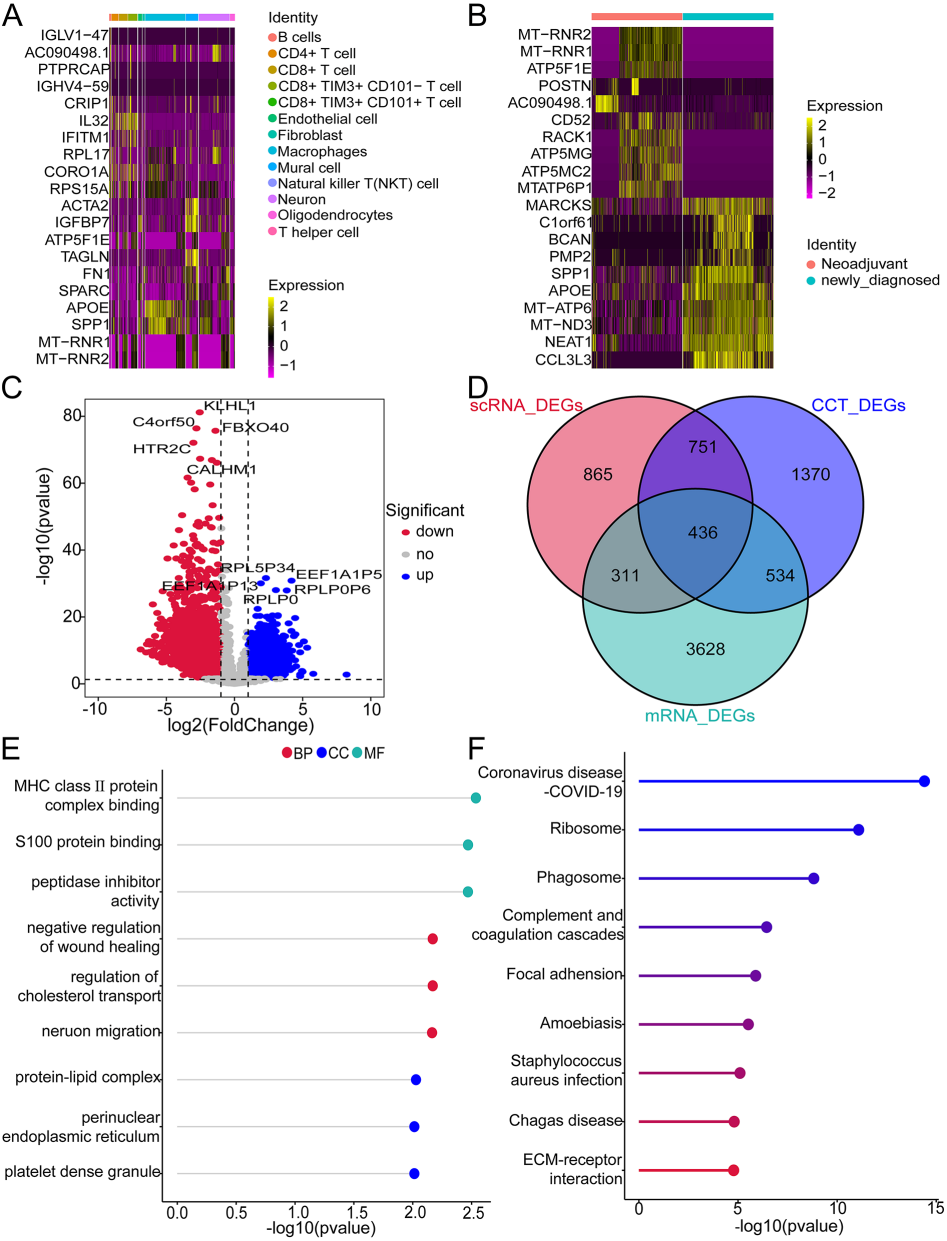
Enrichment analysis of CCT-related genes with differential expression in glioblastoma. (**A**) The heatmap depicts the significantly differentially expressed genes in single-cell glioblastoma CCT. (**B**) The heatmap illustrates the top 20 DEGs with significant differential expression in single-cell newly diagnosed and neoadjuvant therapy groups. (**C**)The heatmap shows 10 DEGs with significant differential expression in the transcriptome of glioblastoma samples compared to normal control samples. (**D**) Venn diagram displaying the intersection genes. (**E**) GO enrichment analysis of key genes. (**F**) KEGG enrichment analysis of key genes

In the single-cell data, there are 3091 differentially expressed genes between CCT and other cell populations. The top 10 upregulated genes (IGLV1-47, AC090498.1, PTPRCAP, IGHV4-59, CRIP1, IL32, IFITM1, RPL17, CORO1A, RPS15A) and top 10 downregulated genes (ACTA2, IGFBP7, ATP5F1E, TAGLN, FN1, SPARC, APOE, SPP1, MT-RNR1, MT-RNR2) in CCT cells were displayed in a heatmap (Table S6, Fig. 7A). Based on the comparison of single-cell data between newly diagnosed and neoadjuvant therapy groups, 2363 DEGs were identified (Table S7). The top 10 upregulated genes (MT-RNR2, MT-RNR1, ATP5F1E, POSTN, AC090498.1, CD52, RACK1, ATP5MG, ATP5MC2, MTATP6P1) and the top 10 downregulated genes (MARCKS, C1orf61, BCAN, PMP2, SPP1, APOE, MT-ATP6, MT-ND3, NEAT1, CCL3L3) from the neoadjuvant therapy group were displayed in a heatmap (Fig. 7B). In addition, by comparing transcriptome data between GBM samples and normal controls, 4909 DEGs were identified (Fig. 7C, Table S8). The intersection of the DEGs from the three groups resulted in 436 intersecting genes (Fig. 7D). In order to understand what functions cross genes perform, we analyzed GO (Table S9) and KEGG (Table S10) pathways for cross genes. According to the GO analysis, these genes are related to biological processes such as “negative wound healing”, “cholesterol transport”, and “neurons migration” cellular components including “protein-lipid complex”, “perinuclear endoplasmic reticulum” and “platelet dense granule”; and molecular functions like “MHC class II protein complex binding” (Fig. 7E). Genes associated with Coronavirus disease - COVID-19 and Phagosome are enriched in KEGG results (Fig. 7F).

### Prognostic model construction and validation

**Fig. 8:**
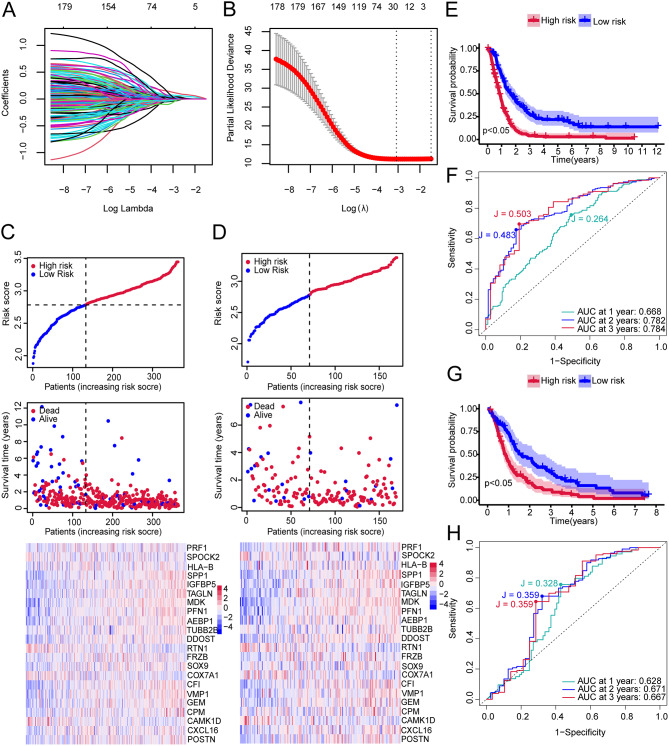
Glioblastoma intersection gene Cox and LASSO regression analysis. (**A**) The trajectory of variable changes in LASSO regression, where the x-axis represents the logarithm of the independent variable lambda and the y-axis represents the coefficients that can be independently obtained. (**B**) Confidence intervals at each lambda in LASSO regression. (**C**)Risk triplot from the training set. (**D**) Risk triplot from the validation set. (**E**) Survival curves for high-risk and low-risk group patients from the training set. Red represents the high-risk group, and blue represents the low-risk group. (**F**) Time-dependent ROC curves for the model in the training set at 1 year, 2 years, and 3 years. (**G**) Survival curves for high-risk and low-risk group patients from the validation set. Red represents the high-risk group, and blue represents the low-risk group. (**H**) Time-dependent ROC curves for the model in the validation set at 1 year, 2 years, and 3 years

Based on the intersecting genes identified previously, a univariate Cox regression analysis revealed 180 genes significantly associated with GBM prognosis (Table S11). LASSO regression analysis then identified 22 prognostic genes in GBM patients (Table S12), as shown in Fig. 8A and B. Figure 8C and D present risk triplets for patient groups in the training and validation cohorts. KM survival analysis indicated significantly worse outcomes for high-risk patients (Fig. 8E&G). ROC analysis confirmed the prediction model’s strong performance in both cohorts (Fig. 8F&H). At the same time, in both cohorts, the 1-year, 2-year, and 3-year Youden’s index values were greater than 0.2. In the training cohort, the 2-year and 3-year Youden’s index values exceeded 0.4, indicating that the model demonstrated a more stable ability to predict the long-term prognosis of patients (Fig. 8F&H). Additionally, we examined the impact of prognostic gene expression levels on outcomes, finding all prognostic genes significantly affected prognosis (Supplementary Figs. 2, 3, 4, and 5). We also discovered that age (high>=55, low < 55) significantly affects prognosis (Supplementary Fig. 6).

### Nomogram construction and validation

**Fig. 9:**
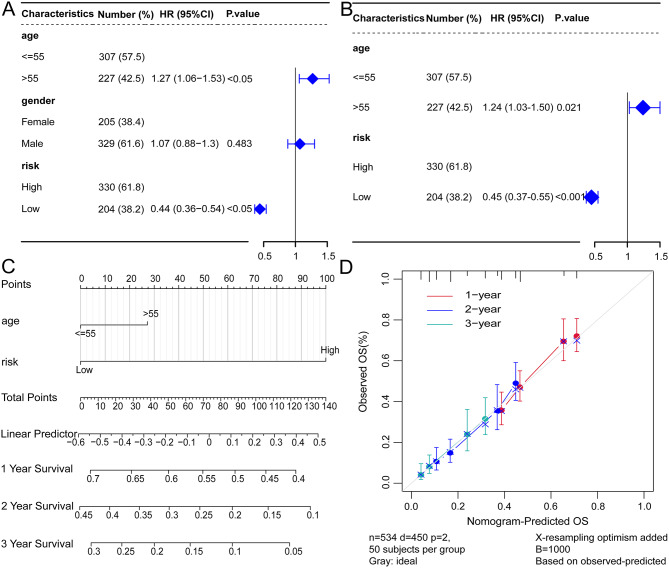
Construction and validation of a nomogram. (**A**) The forest plot displays the results of univariate Cox regression analysis conducted on clinical features. (**B**) The forest plot illustrates the results of multivariate Cox regression analysis conducted on clinical features. (**C**)The nomogram of the prediction model. The segments represent the contribution of clinical factors to the outcome events, the total score represents the overall score of individual values for all variables, and the bottom three lines represent the prognosis for the 1-year, 2-year, and 3-year survival periods corresponding to each value point. (**D**) Calibration curves for 1-year, 2-year, and 3-year survival periods in the nomogram

Subsequently, Cox regression analyses (univariate and multivariate) were performed, integrating clinical characteristics (age and gender) and patients’ risk scores. The findings indicated that the risk score is an independent prognostic factor for glioblastoma multiforme patients (Fig. 9A and B). Figure 9C’s nomogram illustrates that risk scores are significant predictors of GBM patient prognosis. The Calibration curve further revealed that the nomogram exhibited excellent predictive performance for OS in GBM patients (Fig. 9D).

### GESA and GSVA

**Fig. 10:**
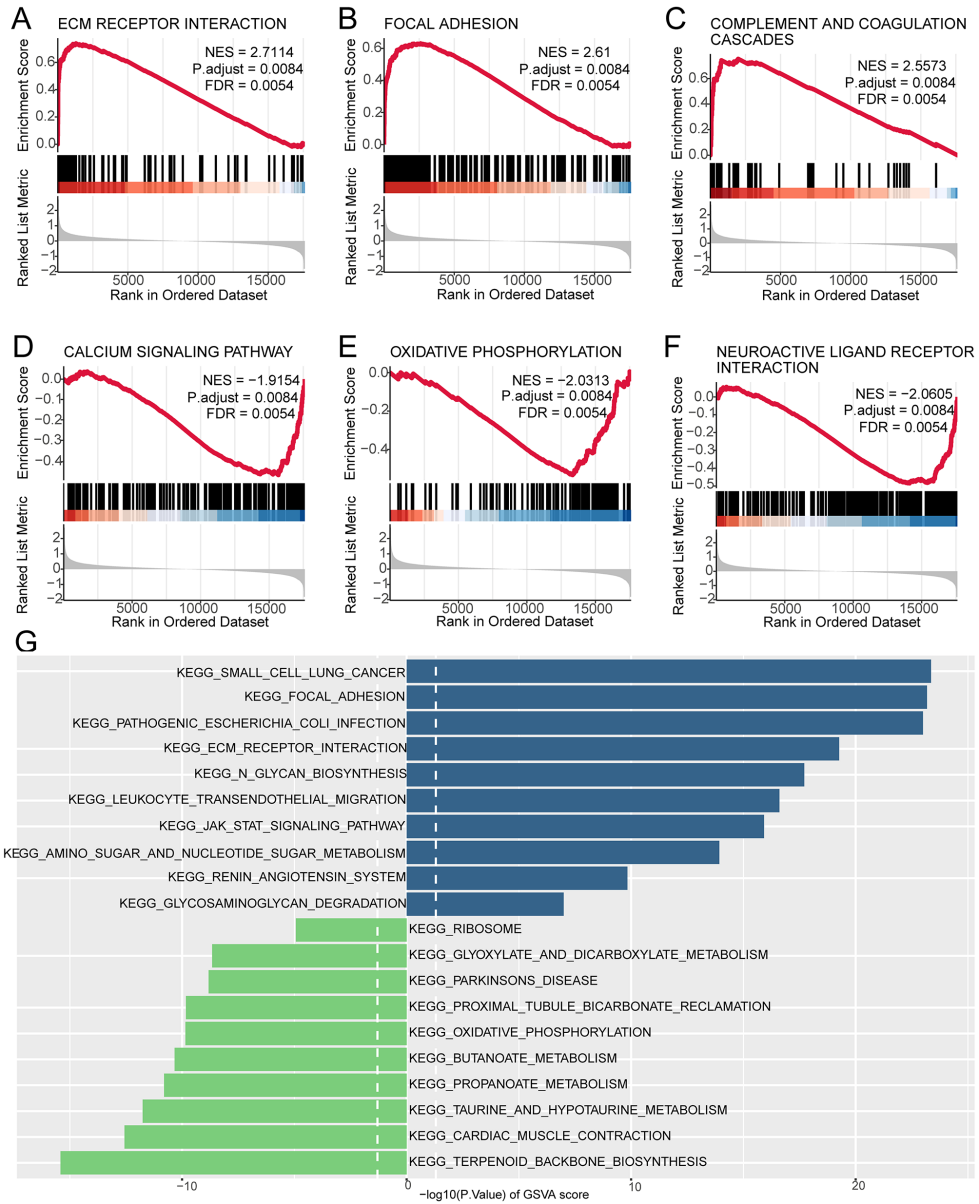
Enriched pathways identified by GSEA and GSVA analyses. GSEA analysis revealed significant enrichment of ECM RECEPTOR INTERACTION (**A**), FOCAL ADHESION (**B**), COMPLEMENT AND COAGULATION CASCADES (**C**), CALCIUM SIGNALING PATHWAY (**D**), OXIDATIVE PHOSPHORYLATION (**E**), and NEUROACTIVE LIGAND RECEPTOR INTERACTION (**F**).(**G**) Bar graph of GSVA enrichment analysis showing differentially enriched pathways between high-risk and low-risk groups

Using GSEA, we explored the potential mechanisms underlying differential gene expression. Based on the normalized enrichment score (NES) of the MsigDB database, the most significant pathways were identified (Table S13). The GSEA results showed significant enrichment of ECM RECEPTOR INTERACTION, FOCAL ADHESION, and COMPLEMENT AND COAGULATION CASCADES in the high-risk group (Fig. 10A-C), while CALCIUM SIGNALING PATHWAY, OXIDATIVE PHOSPHORYLATION, and NEUROACTIVE LIGAND RECEPTOR INTERACTION were significantly enriched in the low-risk group (Fig. 10D-E). Furthermore, GSVA enrichment analysis revealed that pathways such as KEGG_SMALL_CELL_LUNG_CANCER and KEGG_FOCAL_ADHESION were significantly upregulated in the high-risk group. Conversely, pathways like KEGG_TERPENOID_BACKBONE_BIOSYNTHESIS and KEGG_CARDIAC_MUSCLE_CONTRACTION were significantly downregulated (Fig. 10G, Table S14).

### Immune infiltration analysis

**Fig. 11:**
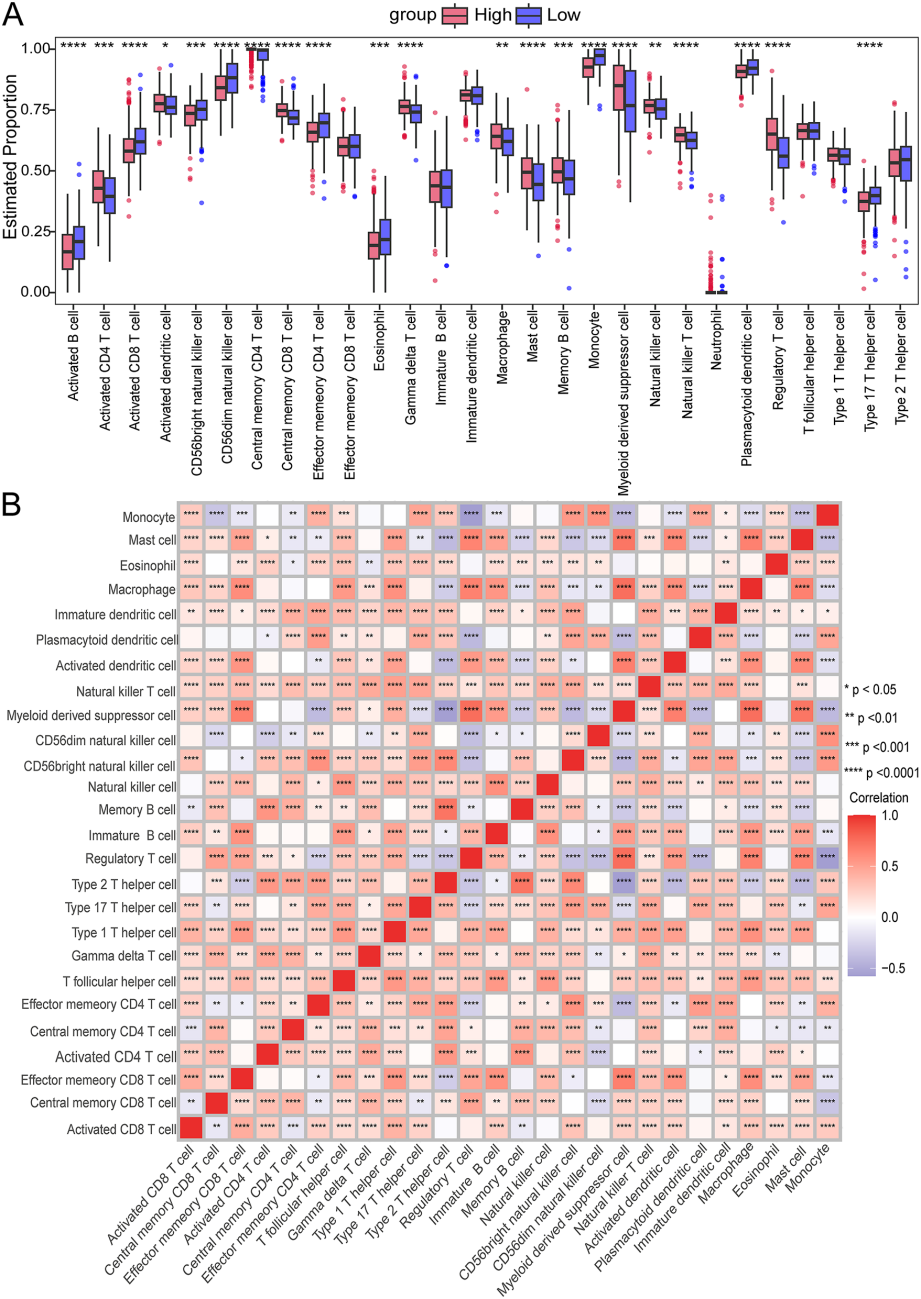
Immune infiltration levels between high-risk and low-risk groups. (**A**) Boxplot showing the estimated proportions of immune cells between high-risk and low-risk groups. (**B**) Correlation between immune cells. Stars denote significance levels: ****p < 0.0001, ***p < 0.001, **p < 0.01, *p < 0.05

**Fig. 12:**
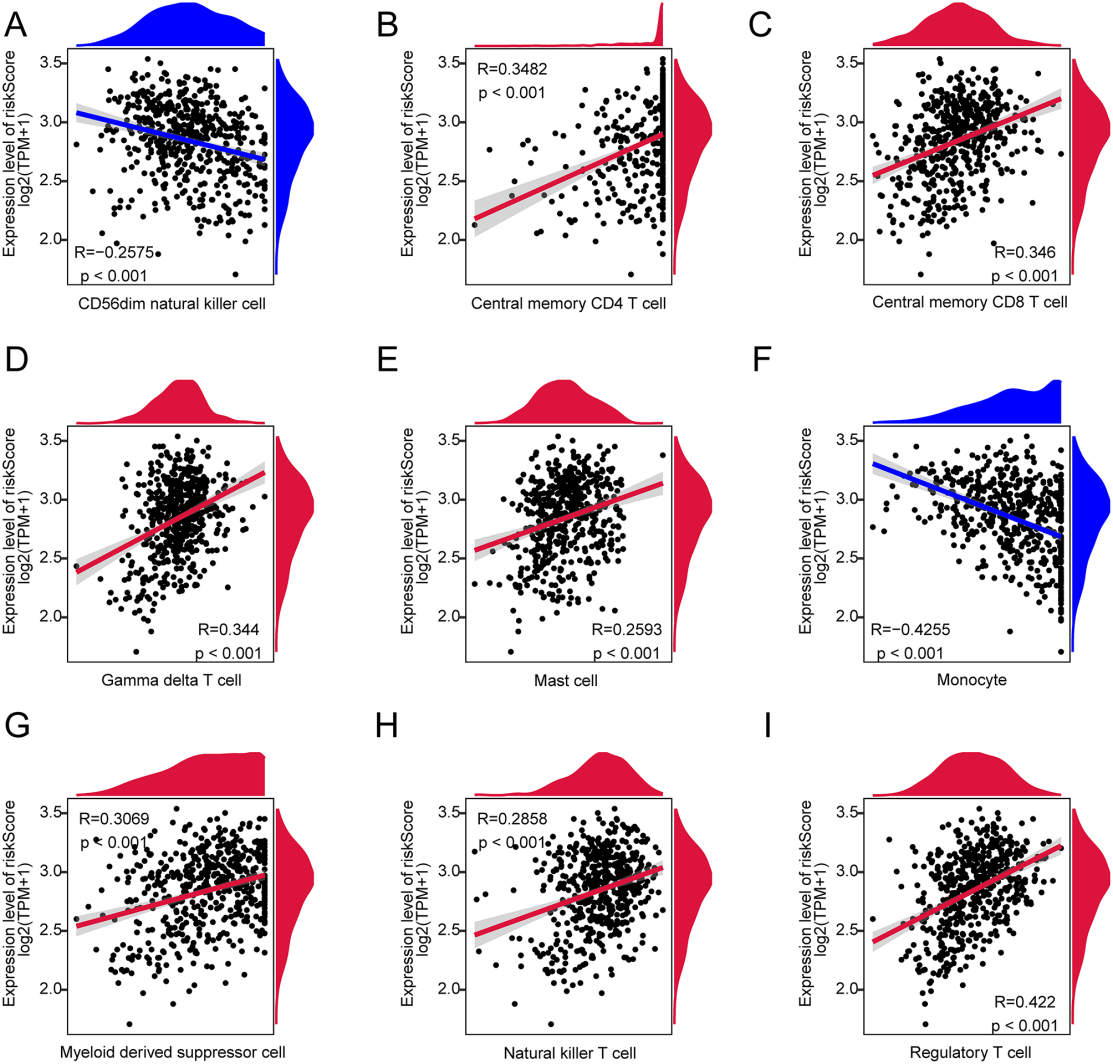
Correlation scatter plots between risk score and immune cells. The correlation scatter plots display the relationship between risk score and (**A**) CD56dim natural killer cell, (**B**) Central memory CD4 T cell, (**C**) Central memory CD8 T cell, (**D**) Gamma delta T cell, (**E**) Mast cell, (**F**) Monocyte, (**G**) Myeloid-derived suppressor cell, (**H**) Natural killer T cell, and (**I**) Regulatory T cell

In high-risk and low-risk GBM groups, we examined 28 immune cell types infiltration levels using the ssGSEA method. Compared to the low-risk group, the high-risk group exhibited significantly reduced infiltration levels of nine types of immune cells, including Activated B cells, Activated CD8 T cells, and CD56bright natural killer cells (Fig. 11A). Conversely, the infiltration levels of twelve types of immune cells, such as Activated CD4 T cells, Activated dendritic cells, and Central memory CD4 T cells, were significantly elevated in the high-risk group relative to the low-risk group (Fig. 11A). There was a significant positive correlation between the majority of immune cells, while negative correlations were observed between a few types of immune cells, such as monocytes and central memory CD8 T cells (Fig. 11B). Figure 12A-I displays the top nine associations between the risk score and immune cell populations. There was a significant positive correlation between a risk score and seven of the nine immune cell types studied, with the exception of the CD56dim natural killer cell and monocytes. These results further underscore the complexity of the immune environment in GBM. Several prognostic genes that contribute to the risk score are associated with various immune cells, leading to an indirect alteration of the overall immune landscape in GBM.

### TMB and drug sensitivity analysis

**Fig. 13:**
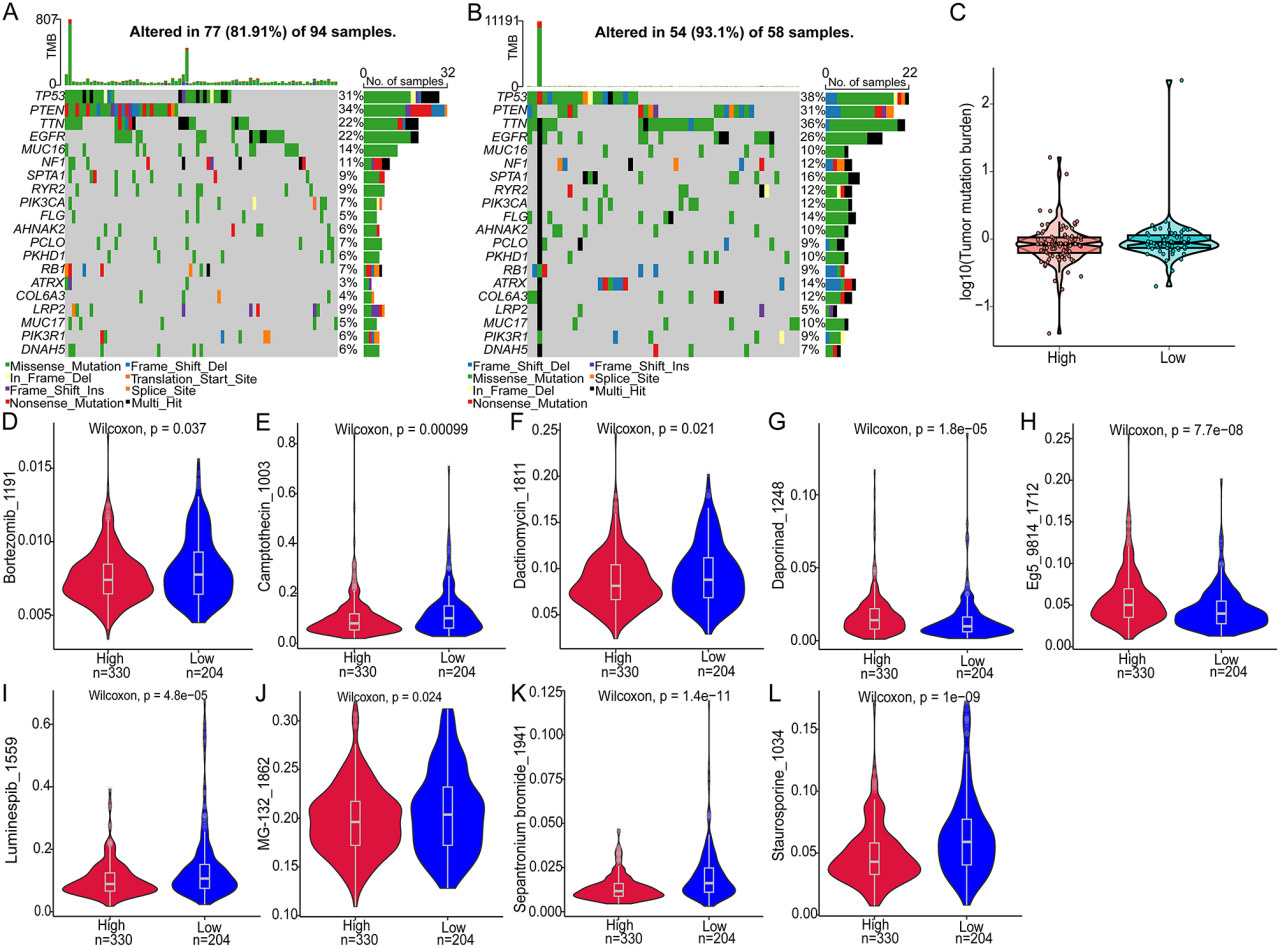
Differences in TMB and drug sensitivity between high-risk and low-risk groups. (**A**) The top 20 genes with the highest mutation frequencies in the high-risk group and (**B**) in the low-risk group. (**C**) Difference in TMB between high-risk and low-risk groups. Violin plots showcasing differences in drug sensitivity between high-risk and low-risk groups, including Bortezomib_1191 (**D**), Camptothecin_1003 (**E**), Dactinomycin_1811 (**F**), Daporinad_1248 (**G**), Eg5_9814_1712 (**H**), Luminespib_1559 (**I**), MG-132_1862 (**J**), Sepantronium bromide_1941 (**K**), and Staurosporine_1034 (**L**) are illustrated

A list of the top 20 genes with the highest mutation frequency was developed in order to assess the specific gene mutations in glioblastoma. Both groups showed that TP53 mutations were the most common, followed by PTEN mutations (Fig. 13A and B). TMB is a crucial criterion for immunotherapy success. Therefore, we examined somatic mutations associated with glioblastoma and found that high-risk and low-risk groups did not differ significantly in TMB (Fig. 13C). Furthermore, an analysis was performed to evaluate the predictive utility of the risk score in determining chemotherapy sensitivity among patients with GBM (Table S15). High-risk patients exhibited greater sensitivity to Bortezomib_1191 (Fig. 13D), Camptothecin_1003 (Fig. 13E), Dactinomycin_1811 (Fig. 13F), Luminespib_1559 (Fig. 13I), MG-132_1862 (Fig. 13J), Sepantronium bromide_1941 (Fig. 13K), and Staurosporine_1034 (Fig. 13L). Therefore, chemotherapy might be a good choice for those with high-risk scores.

### TIDE and immune checkpoints analysis

**Fig. 14:**
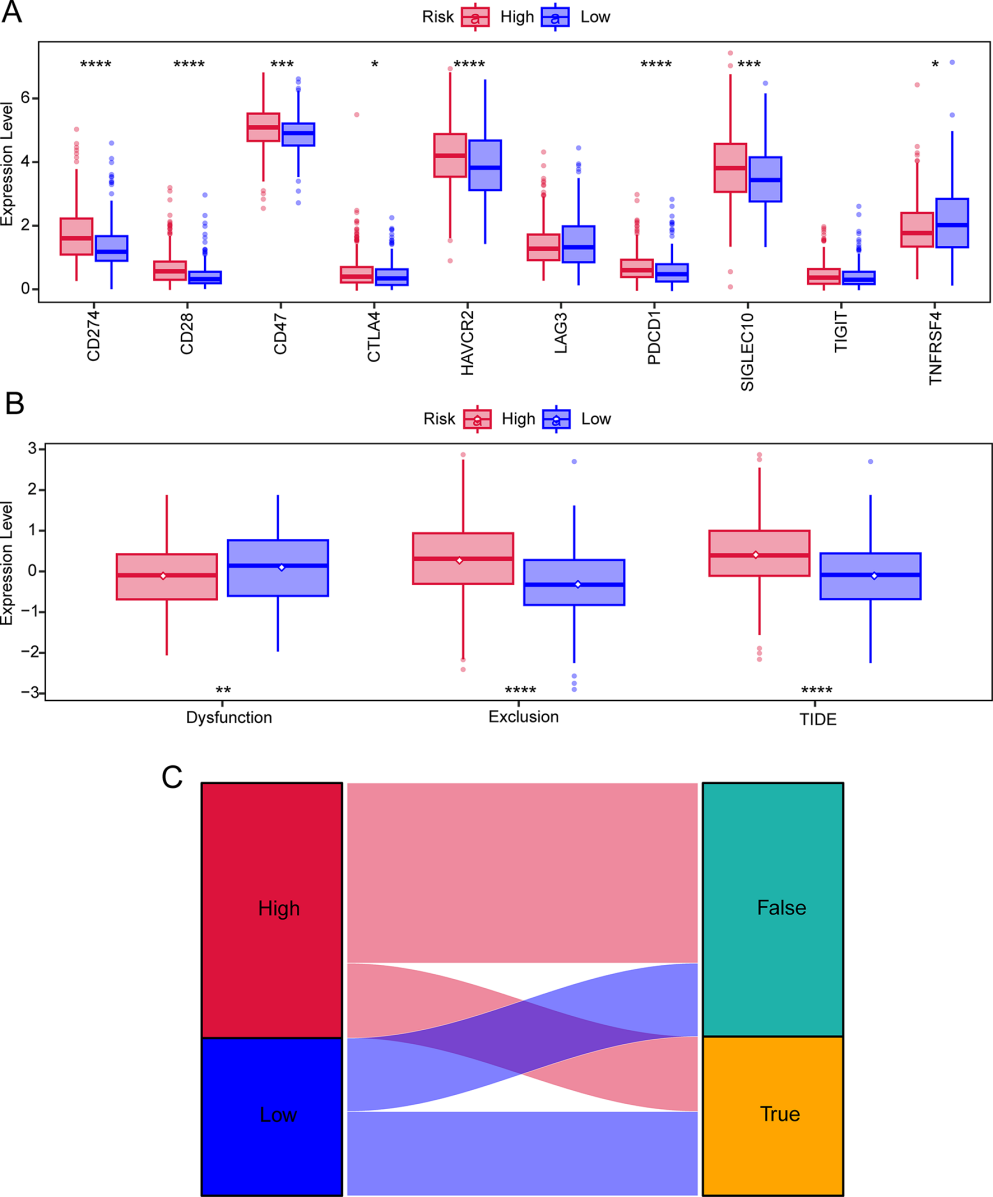
TIDE and immune checkpoint analysis. (**A**) Boxplot showing the level differences of immune checkpoints between high-risk and low-risk groups. (**B**) Differences in T cell dysfunction, T cell exclusion, and TIDE score between high-risk and low-risk groups. (**C**)Sankey diagram illustrating the predicted response to immunotherapy based on TIDE analysis

Our study examined the differential expression of immune checkpoints between high-risk and low-risk individuals, revealing significant expression level differences in CD274, CD28, CD47, CTLA4, HAVCR2, PDCD1, SIGLEC10, and TNFRSF4 between the two risk groups (Fig. 14A). In both low-risk and high-risk groups, TIDE was used to assess the potential clinical efficacy of immunotherapy (Table S16). According to our study results, low-risk individuals scored significantly lower on TIDE and excluded significantly less than high-risk individuals, while high-risk individuals scored significantly higher on Dysfunction (Fig. 14B). Moreover, most immunotherapy non-responses were reported in high-risk patients (Fig. 14C).

## Discussion

Among malignant brain tumors, GBM has a high incidence and low survival rate [1]. Currently, there is an abundance of preclinical and clinical research on GBM. However, the complex infiltrative and heterogeneous nature of GBM impedes curative treatments, increases disease recurrence, and leads to the poor patient prognosis. Therefore, identifying new biological targets and developing innovative diagnostic and therapeutic strategies are crucial for enhancing the prognosis of patients with GBM. This study aims to assess the prognostic significance of DEGs associated with CCT cells in GBM and their function in the immune microenvironment using single-cell RNA sequencing, whole-genome expression profiles, and clinical data.

Based on our study, we found that the differentiation process from CD8 + TIM3 + CD101-T cells to CD8 + TIM3 + CD101 + T (CCT) cells revealed distinct gene expression profiles, marking various states along the differentiation trajectory. This discovery provided crucial insights into the dynamic T cell differentiation dynamics within the GBM microenvironment. Enrichment of GO pathways such as “response to unfolded protein” in different transcriptional states indicates their significance in the transition towards mature CCT cells. Unraveling these pathways may unveil novel therapeutic targets for modulating T cell differentiation and functionality. Furthermore, the identification of key regulatory genes during this transition presents potential biomarkers for monitoring disease progression and treatment response.

Cell communication revealed significant changes in CCT cells before and after immunotherapy, with a focus on the signals received by CD8 + TIM3 + CD101-T cells, as they may be key drivers of their differentiation. Overall, post-PD-1 blockade therapy showed a slight increase in the number of communication signals associated with CD8 + TIM3 + CD101-T cells, but a marked decrease in overall signal intensity, with communication primarily shifting towards CD8 + TIM3 + CD101 + T cells, representing the major responders in the immune response outcomes. In particular, we observed a significant decrease in the SPP1 communication signals received from the range of cell types, including macrophages, fibroblasts, and endothelial cells, after immunotherapy. Previous studies have reported that the SPP1 + Macrophage-Fibroblast structure in hepatocellular carcinoma can inhibit the efficacy of PD-1 blockade therapy [28, 29]. Additionally, Jin Xing et al. found that Macrophages in glioblastoma can regulate a complex cell communication network through the SPP1-CD44 axis, influencing the immune suppression in the tumor microenvironment [30]. Moreover, studies suggested that SPP1 in GBM sustains GBM survival and stimulates tumor angiogenesis by upregulating the expression of PSMA in endothelial cells through the transcription factor HIFα [31, 32]. Therefore, it is hypothesized that the reduction in SPP1 pathway communication from Macrophages, Fibroblasts, and Endothelial cells to CD8 + TIM3 + CD101-T cells following ICI treatment may be a crucial checkpoint driving the differentiation of CCT precursors into terminal CCT cells, highlighting the significance of SPP1 pathway in immunotherapy.

We established a prognostic model identifying 22 genes significantly correlated with GBM prognosis. Within this model, CAMK1D, COX7A1, RTN1, and SPOCK2 exhibited protective effects, while other genes conferred adverse prognostic analysis. These genes have been linked to tumorigenesis and development. Lung cancer patients with reduced expression of CAMK1D, an inhibitory kinase in the calcium/calmodulin-dependent protein kinase family, have a poorer prognosis [33, 34]. COX7A1, a subunit of cytochrome c oxidase, may influence the clinical efficacy of immunotherapy in gastric cancer patients by promoting M2 macrophage differentiation and inhibiting M1 macrophage differentiation [35]. For neurologic disorders and cancer, RTN1, an endoplasmic reticulum-related Reticulon gene, may serve as a diagnostic/therapeutic biomarker [36]. Glycoprotein SPOCK2, involved in extracellular matrix formation, is critical for cell invasion and metastasis in cancer. Upregulated SPOCK2 in endometrial cancer can suppress tumor cell invasion and migration by modulating MMP2 activation [37]. AEBP1 is a transcriptional repressor factor associated with the pathogenesis of various cancers, including thyroid cancers and breast cancers [38, 39]. Multiple cancer types exhibit aberrant expression of chemokine ligand 16 (CXCL16), which serves as a prognostic factor and indicator of tumor progression [40, 41]. DDOST encodes a non-catalytic subunit of the dolichyl-diphosphooligosaccharide-protein glycosyltransferase complex, which undergoes protein N-glycosylation. There is a strong link between high DDOST expression in glioblastoma patients and a poor prognosis, and DDOST expression is closely correlated with an immunosuppressive microenvironment in GBM patients [42]. IGFBP5 acts as a ligand for ROR1. It has been identified as a promising target in GBM stem-like cells (GSCs) invasion, promoting GSC invasion and tumorigenesis via the ROR1/HER2-CERB signaling axis [43]. The specific genes highlighted in the model underscore their potential as therapeutic targets. Furthermore, the influence of clinical parameters such as age and gender on prognosis underscored the importance of personalized treatment approaches.

The excellent performance of the prognostic prediction model has sparked our interest in exploring the potential mechanisms of differential gene expression. According to GSEA and GSVA analyses, GBM patients at high risk have significant enrichment of ECM RECEPTOR INTERACTION, supporting previous studies emphasizing ECM’s critical role in the progression of GBM. In contrast to other parts of the body, the ECM of the nervous system is primarily composed of proteins and polysaccharides, forming a complex structural and biochemical network [44]. In GBM, the ECM not only supports tumor structure but also influences cellular behaviors, including migration, invasion, and resistance to treatment [45]. Additionally, several other enriched signaling pathways are related to immune regulation, including FOCAL ADHESIONS, LEUKOCYTE TRANSENDOTHELIAL MIGRATION, and JAK STAT SIGNALING [46–48]. The poor prognosis observed in high-risk patients could be partially linked to disrupted anti-tumor immune regulation.

Several immune cells types were significantly different between high-risk and low-risk groups in the immune infiltration analysis. Interestingly, the high-risk group had fewer CD8 and B cells activated than the low-risk group, but more CD4 and central memory CD4 T cells. Researchers have reported similar findings in previous studies, indicating decreased CD8 T cells and activated B cells in tumor microenvironments impede tumor progression [49, 50]. It was found that tumors contained high concentrations of activated CD4 T cells and central memory CD4 T cells, which were able to exert anticancer effects [51–53]. Furthermore, the positive correlations between most immune cells and the negative correlation between monocytes and central memory CD8 T cells highlight the interplay within the tumor microenvironment. In light of these findings, it is evident that the immune microenvironment plays a crucial role in the progression and prognosis of GBM. In addition, immune checkpoint inhibitors (ICIs) can effectively eradicate tumor cells by activating the tumor immune response [54, 55]. Therefore, identifying GBM biomarkers that may influence patient responses to ICIs is crucial. Significant variance was observed in the expression levels of eight checkpoint markers, encompassing CD274 and CD28. Furthermore, predictions from the TIDE algorithm regarding the effectiveness of immune therapy indicated a higher likelihood of benefit for low-risk patients.

Considering that chemoresistance is one of the primary obstacles in GBM treatment [56], distinguishing individuals sensitive to chemotherapy can significantly enhance the therapeutic outcomes of GBM. Our study identified heightened sensitivity to eight drugs, including Bortezomib_1191, Camptothecin_1003, and Dactinomycin_1811, in patients classified within the high-risk GBM group. This finding offered new insights into the treatment strategies for the high-risk group.

## Limitations

Firstly, the absence of wet-lab experimental validation constrains the direct applicability of our findings. Although our bioinformatics analysis offers valuable insights, experimental confirmation is imperative to substantiate the identified gene expressions and pathways. Secondly, our sample size is relatively small, which could affect generalizability. Additionally, the absence of multi-center data validation raises concerns about potential batch effects and biases inherent to single-center datasets. Finally, it is essential to recognize that acquiring data from patients exhibiting diverse responses to PD-1 blockade therapy could enable a more precise delineation of the gene and cellular alterations induced by this treatment. Such insights would significantly enhance our understanding of immunotherapy in patients with GBM. This will be a central focus of the subsequent phase of our research.

## Conclusion

Our comprehensive bioinformatics analysis confirmed that CD8 + TIM3 + CD101 + T cells significantly influence the immunological landscape of GBM. The identification of DEGs and the development of a prognostic model have provided new insights into GBM’s molecular mechanisms and identified potential therapeutic targets. These findings contribute to a deeper understanding of the intricate immune microenvironment in GBM and provide valuable insights into PD-1 blockade therapy for GBM patients, potentially improving patient outcomes.

## Electronic supplementary material

Below is the link to the electronic supplementary material.

Supplementary Material 1

Supplementary Material 2

Supplementary Material 3

Supplementary Material 4

Supplementary Material 5

Supplementary Material 6

Supplementary Material 7

Supplementary Material 8

Supplementary Material 9

Supplementary Material 10

Supplementary Material 11

Supplementary Material 12

Supplementary Material 13

Supplementary Material 14

Supplementary Material 15

Supplementary Material 16

## Data Availability

The datasets utilized in this study are accessible upon request from the corresponding author.
