# Supplementary figures and images for: Characterization and prognostic of CD8 + TIM3 + CD101 + T cells in glioblastoma multiforme
Source: Cell Biosci. 2025 May 15;15:60. doi: 10.1186/s13578-025-01390-1 (PMC12083040; doi:10.1186/s13578-025-01390-1)

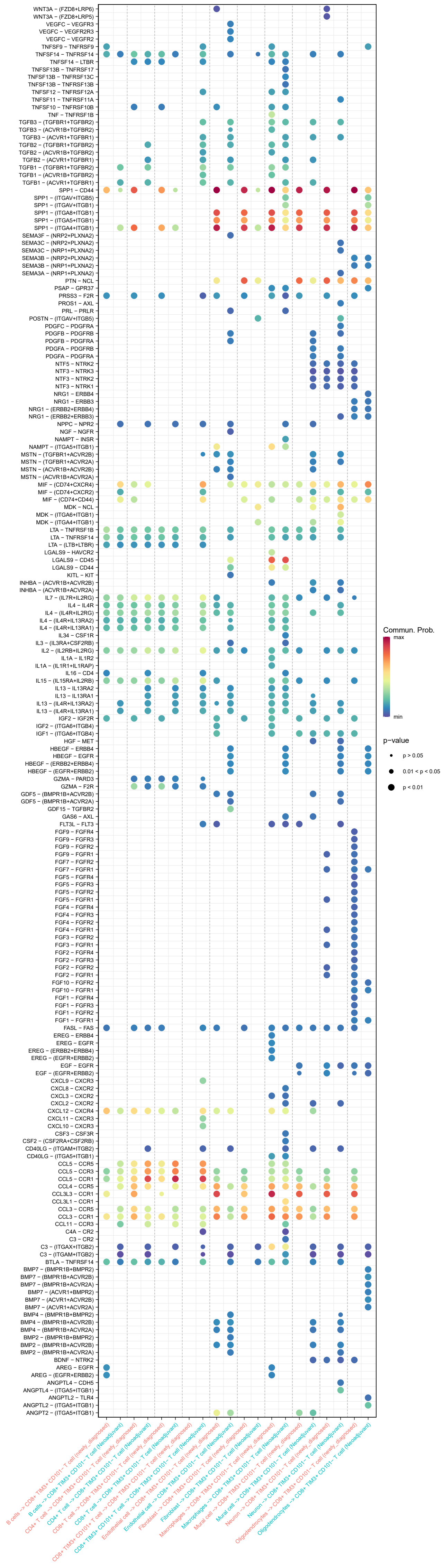

Supplement: Supplementary file 15 — Supplementary Material 15 [file 13578_2025_1390_MOESM15_ESM.pdf]
