# Supplementary material for: Characterization and prognostic of CD8 + TIM3 + CD101 + T cells in glioblastoma multiforme
Source: Cell Biosci. 2025 May 15;15:60. doi: 10.1186/s13578-025-01390-1 (PMC12083040; doi:10.1186/s13578-025-01390-1)
Supplement: Supplementary file 16 — Supplementary Material 16 [file 13578_2025_1390_MOESM16_ESM.docx]

**Supplementary Figures**

**Supplementary Figure 1.** Bubble plot showing the ligand-receptor pairs in the newly diagnosed and neoadjuvant therapy groups.

**
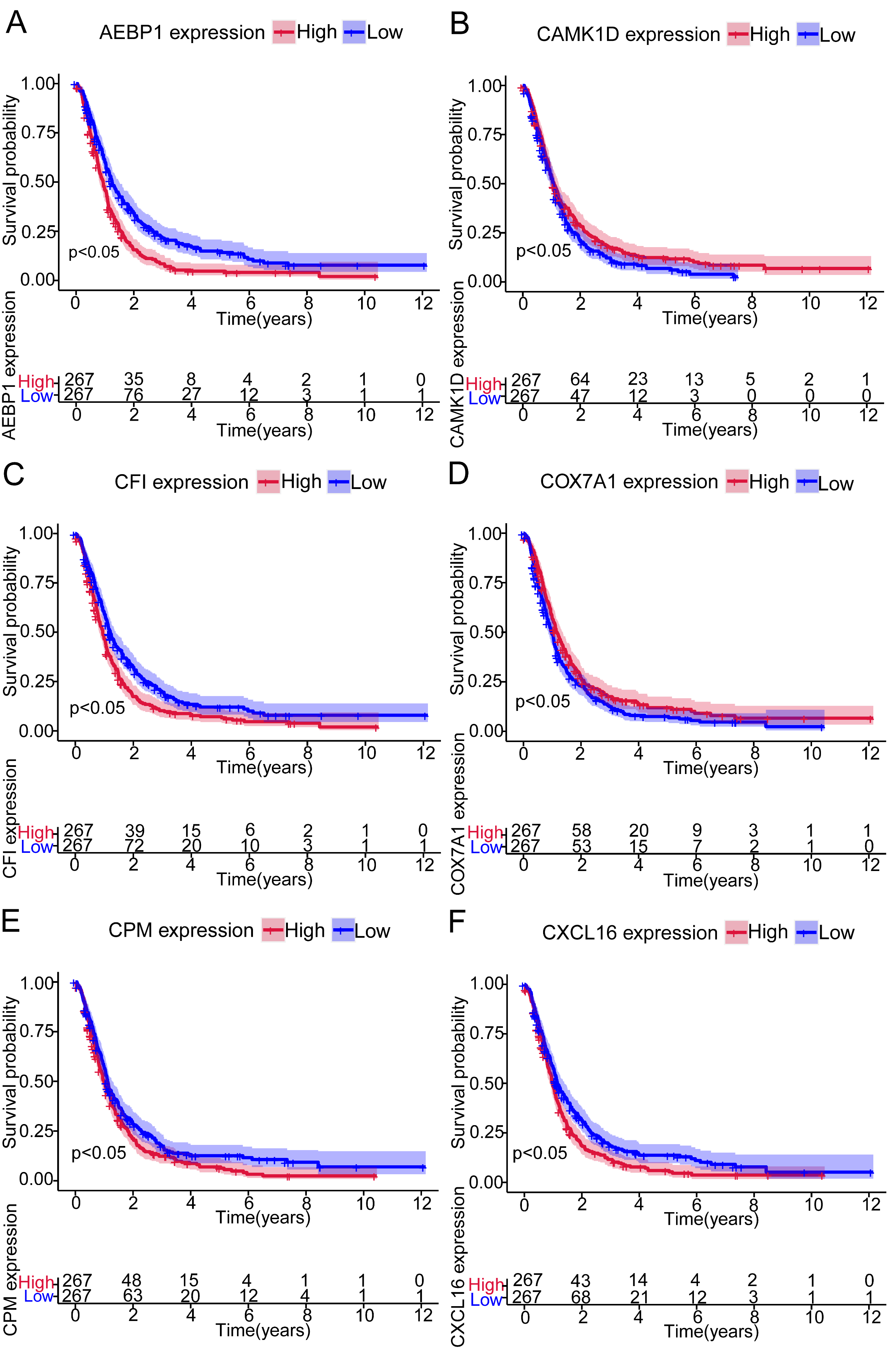
**

**Supplementary Figure 2.** The impact of prognostic gene expression levels on outcomes. (A) AEBP1. (B) CAMK1D. (C) CFI. (D) COX7A1. (E) CPM. (F) CXCL16.


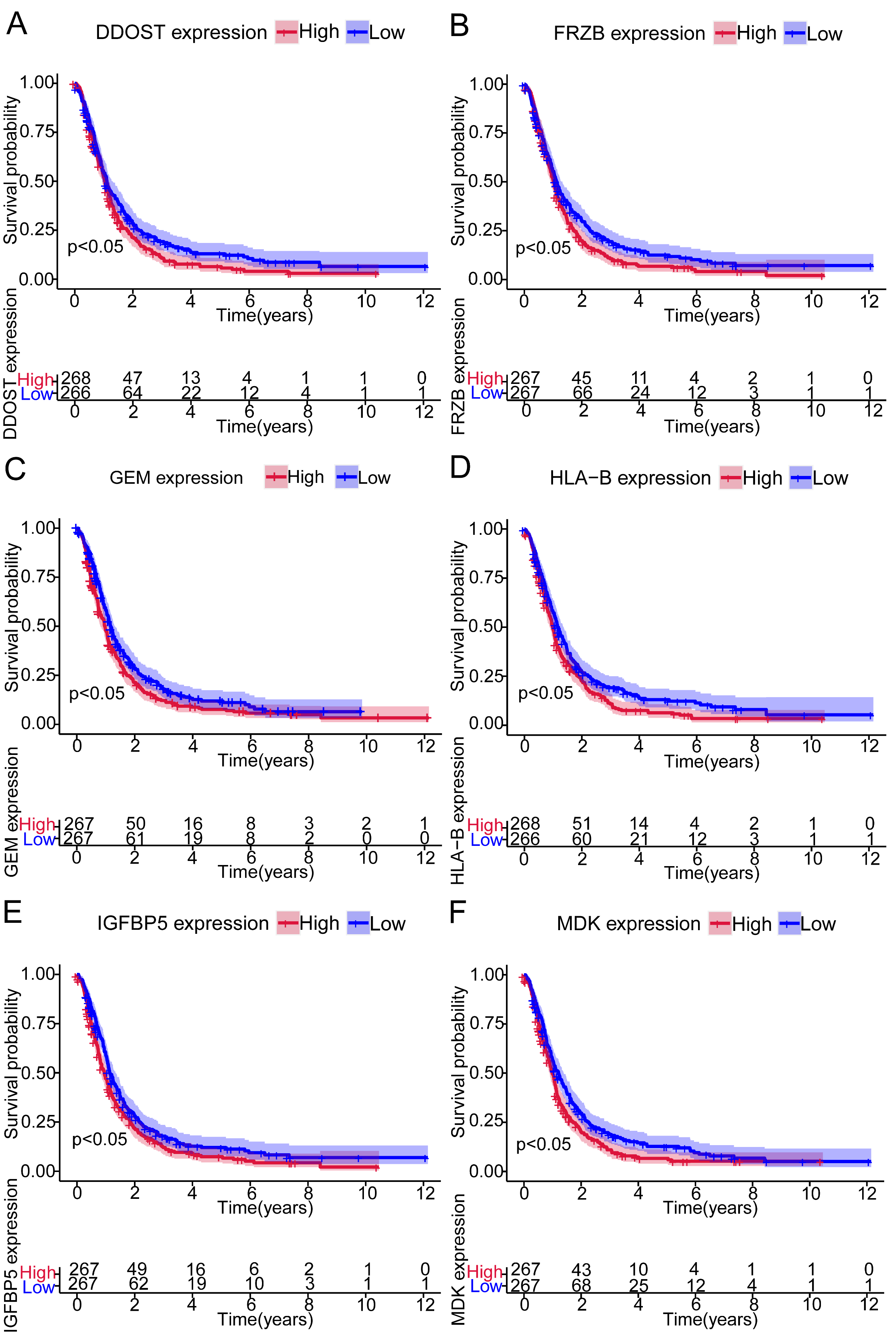


**Supplementary Figure 3.** The impact of prognostic gene expression levels on outcomes. (A) DDOST. (B) FRZB. (C) GEM. (D) HLA-B. (E) IGFBP5. (F) MDK.


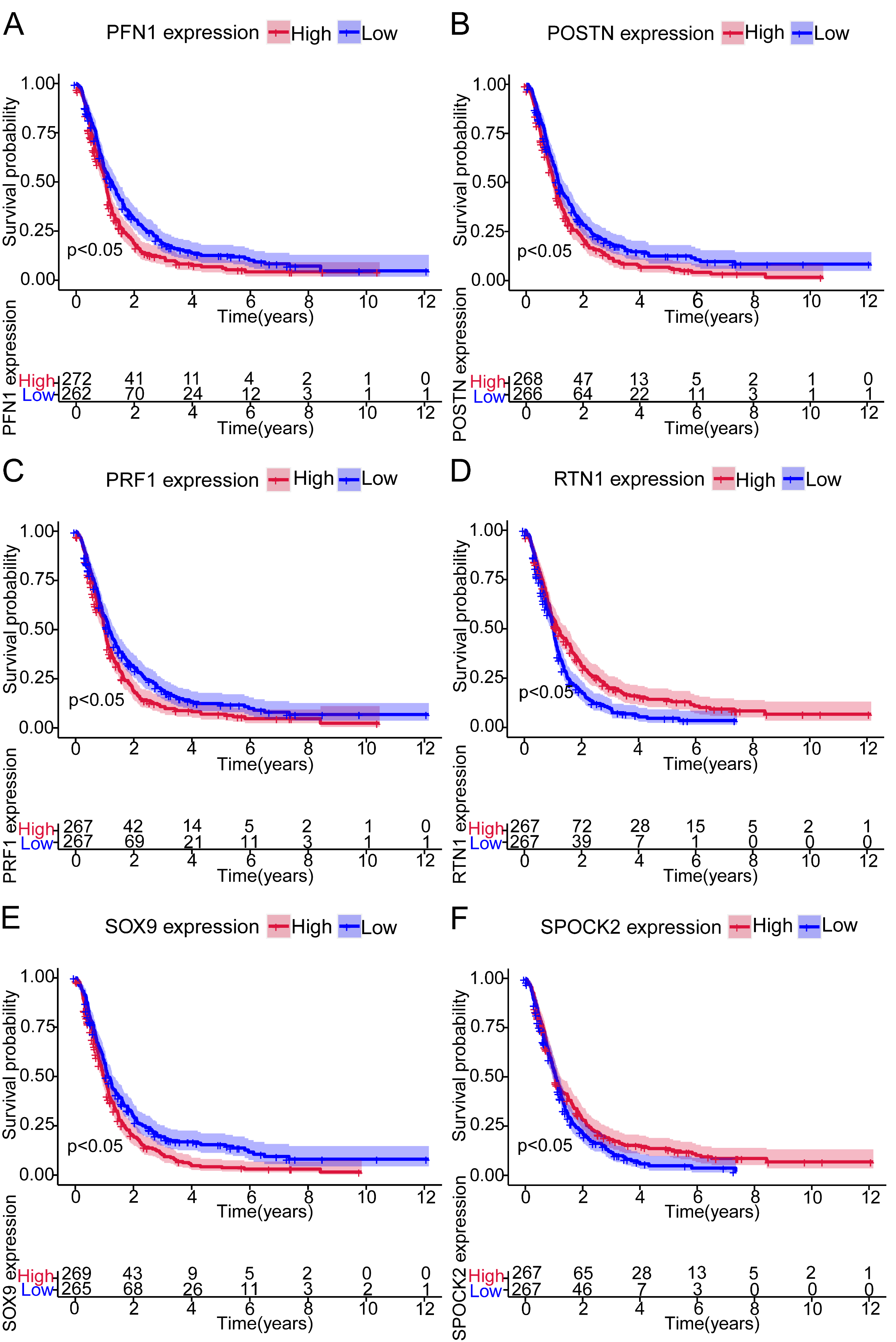


**Supplementary Figure 4.** The impact of prognostic gene expression levels on outcomes. (A) PFN1. (B) POSTN. (C) PRF1. (D) RTN1. (E) SOX9. (F) SPOCK2.


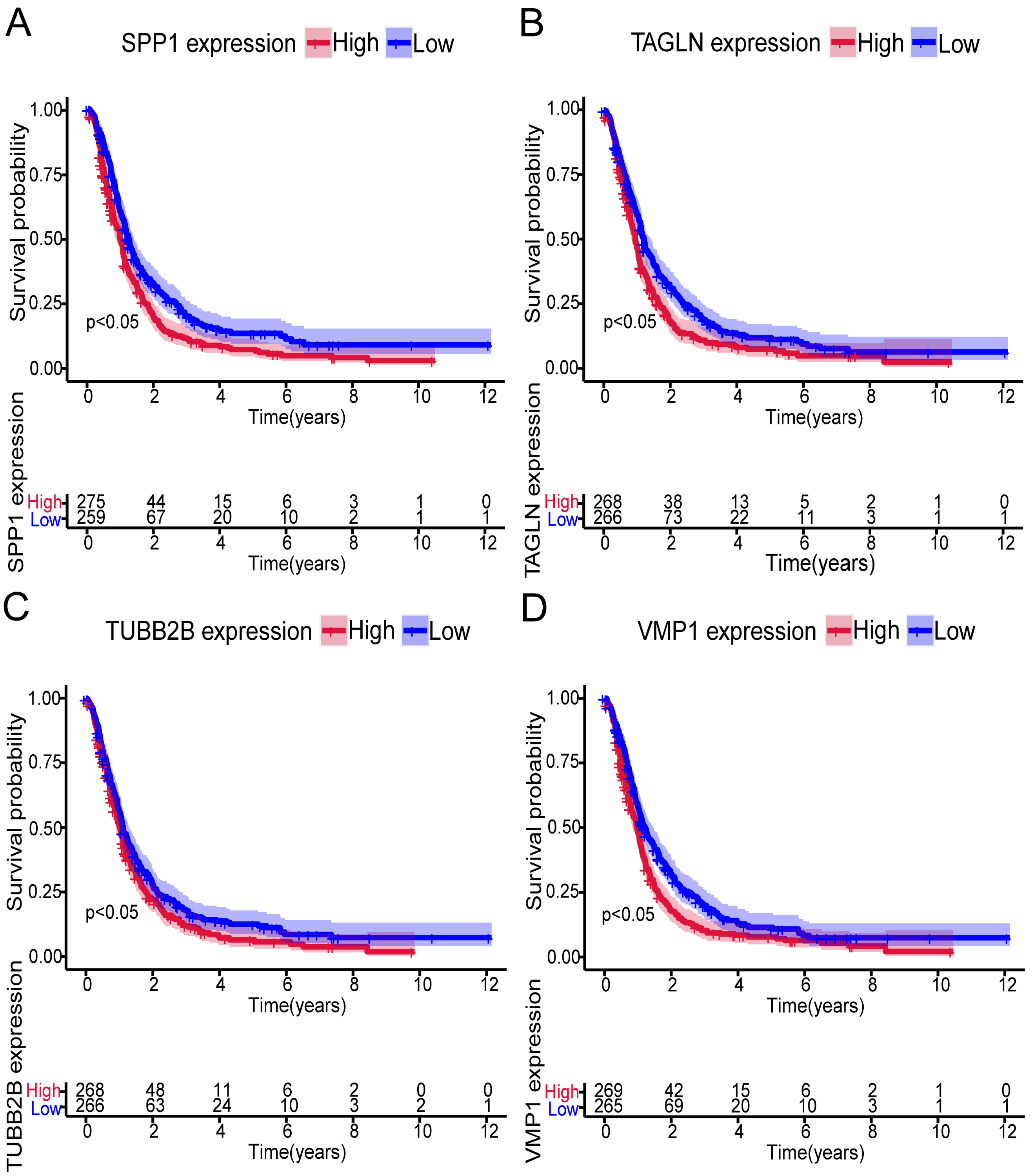


**Supplementary Figure 5.** The impact of prognostic gene expression levels on outcomes. (A) SPP1. (B) TAGLN. (C) TUBB2B. (D) VMP1.


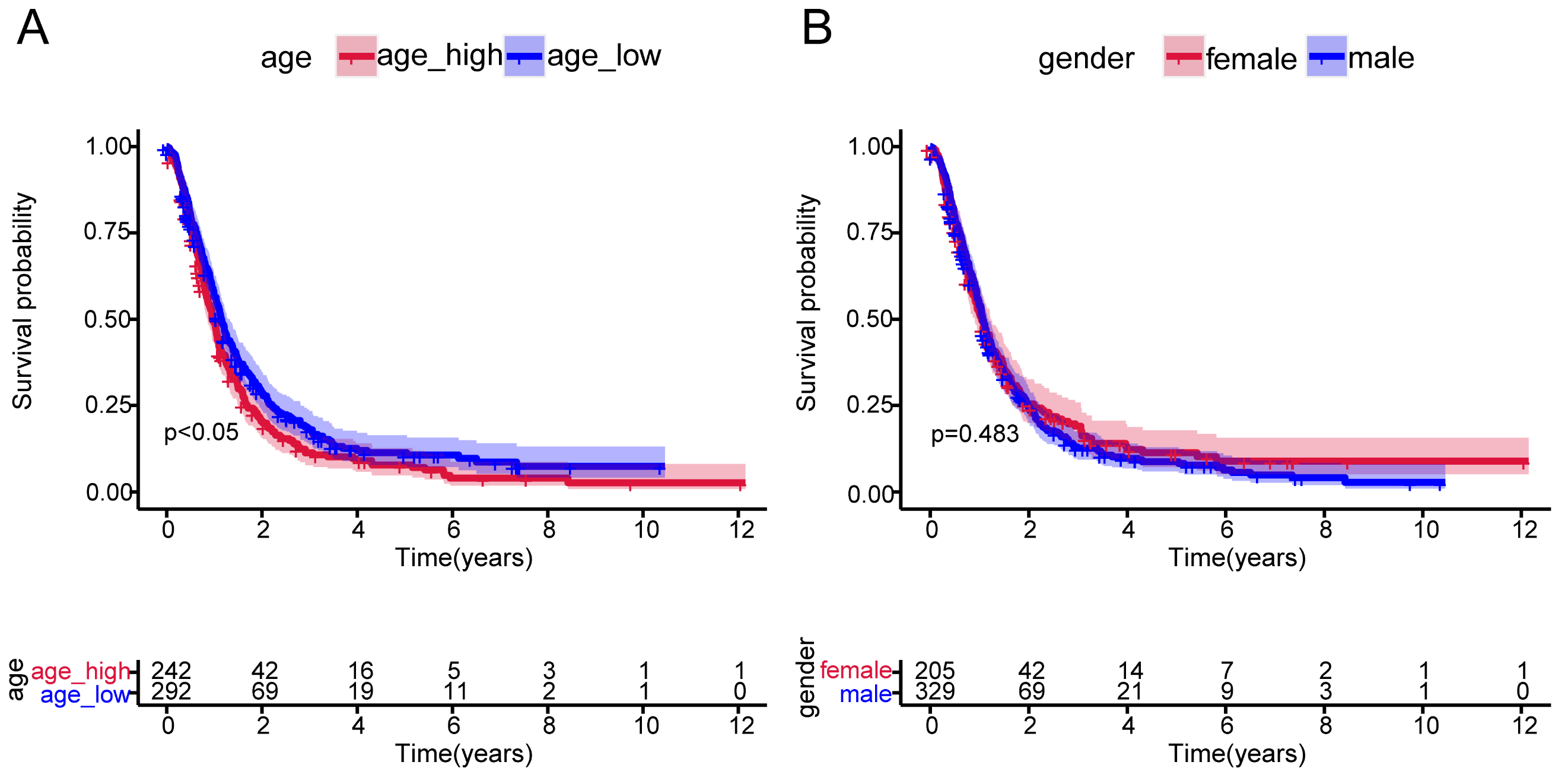


**Supplementary Figure 6.** Impact of various clinical features on prognosis. (A) Age (high＞=55, low<55). (B) Gender.

**Supplementary Tables**

TableS1 Marker genes specific to various cell types

| Cell type | Marker |
| --- | --- |
| T cells | PTPRC, CD3D, CD3E |
| B cells | CD79A, CD19, MS4A1 |
| Macrophages | CD68, CD163, AIF1 |
| Oligodendrocytes | MBP, MOG, MAG |
| Neuron | MAP2, STMN2, GAD2 |
| Mural cell | CD248, FOXF2 |
| Fibroblast | LUM |
| Endothelial cell | CLDN5, FLT1 |

TableS2 The distribution of various cell types across distinct groups

| Samples | Cell type | Cell num | Group num | Cell ratio | Cell percent |
| --- | --- | --- | --- | --- | --- |
| Neoadjuvant | T cells | 24140 | 89958 | 0.268347 | 26.83% |
| Neoadjuvant | Macrophages | 17468 | 89958 | 0.19418 | 19.42% |
| Neoadjuvant | Neuron | 27656 | 89958 | 0.307432 | 30.74% |
| Neoadjuvant | Mural cell | 10812 | 89958 | 0.120189 | 12.02% |
| Neoadjuvant | Endothelial cell | 3089 | 89958 | 0.034338 | 3.43% |
| Neoadjuvant | Oligodendrocytes | 2522 | 89958 | 0.028035 | 2.80% |
| Neoadjuvant | Fibroblast | 3002 | 89958 | 0.033371 | 3.34% |
| Neoadjuvant | B cells | 1269 | 89958 | 0.014107 | 1.41% |
| newly_diagnosed | T cells | 3380 | 23488 | 0.143903 | 14.39% |
| newly_diagnosed | Macrophages | 10856 | 23488 | 0.462193 | 46.22% |
| newly_diagnosed | Neuron | 5099 | 23488 | 0.21709 | 21.71% |
| newly_diagnosed | Mural cell | 1668 | 23488 | 0.071015 | 7.10% |
| newly_diagnosed | Endothelial cell | 1245 | 23488 | 0.053006 | 5.30% |
| newly_diagnosed | Oligodendrocytes | 760 | 23488 | 0.032357 | 3.24% |
| newly_diagnosed | Fibroblast | 196 | 23488 | 0.008345 | 0.83% |
| newly_diagnosed | B cells | 284 | 23488 | 0.012091 | 1.21% |
